# Polysialic acid regulates glomerular microvasculature formation by interaction with VEGF-A188 in mice

**DOI:** 10.1007/s10456-025-09984-6

**Published:** 2025-05-24

**Authors:** Kristina M. Niculovic, Manuel M. Vicente, Vanessa Wittek, Elina Kats, Iris Albers, Kerstin Flächsig-Schulz, Ulrike Peters-Bernard, Anna-Carina Weiss, Hauke Thiesler, Laura S. Dräger, Manuel H. Taft, Anne Jörns, Hans Bakker, Herbert Hildebrandt, Martina Mühlenhoff, Birgit Weinhold, Markus Abeln, Anja K. Münster-Kühnel

**Affiliations:** 1 Institute of Clinical Biochemistry, Hannover Medical School, Carl-Neuberg-Str. 1, 30625 Hannover, Germany; 2 Institute for Biophysical Chemistry, Hannover Medical School, Carl-Neuberg-Str. 1, 30625 Hannover, Germany

**Keywords:** VEGF-A, Sialic acid, Polysialic acid, Kidney development, Microvasculature

## Abstract

Vascular endothelial growth factor A (VEGF-A) is a key signalling protein that stimulates blood vessel development and repair. Its tight control is essential for organ development and tissue homeostasis. However, the complex regulatory network for balanced bioavailability of VEGF-A is not fully understood. Here, we assessed the role of the glycocalyx component polysialic acid (polySia) for kidney development and its potential interactions with VEGF-A isoforms, in vitro and in vivo, using mouse models of polySia deficiency. PolySia acts as negative regulator of cell adhesion, but also may interact with extracellular components. In murine kidney, polySia was identified on nephron progenitor and endothelial cell subsets in developing nephrons with declining expression during maturation. Loss of polySia in *Ncam*^*−/−*^ mice revealed the neural cell adhesion molecule NCAM as major protein carrier. Both polysialyltransferase-negative and *Ncam*^*−/−*^ mice displayed impaired glomerular microvasculature development with reduced endothelial cell numbers, reminiscent to the phenotype of mice with impaired VEGF-A signalling. In vitro, immobilized polySia specifically interacted with the VEGF-A188 isoform demonstrating an isoform-specific direct interaction. Single cell RNA sequencing data analysis of newborn mouse kidneys implicated activation of VEGF-A-signalling in polysialyltransferase-positive endothelial cells. Consistently, loss of polySia resulted in diminished VEGFR2 activation in perinatal kidney and human endothelial cells. At transcriptional level, the expression of polysialyltransferases and known polySia carrier proteins is conserved in human developing kidney. Together, these data demonstrate a direct impact of polySia on VEGF-A signalling with the perspective that polysialylation could be a therapeutic target to ameliorate microvasculature repair after renal injury.

**Graphical abstract:**

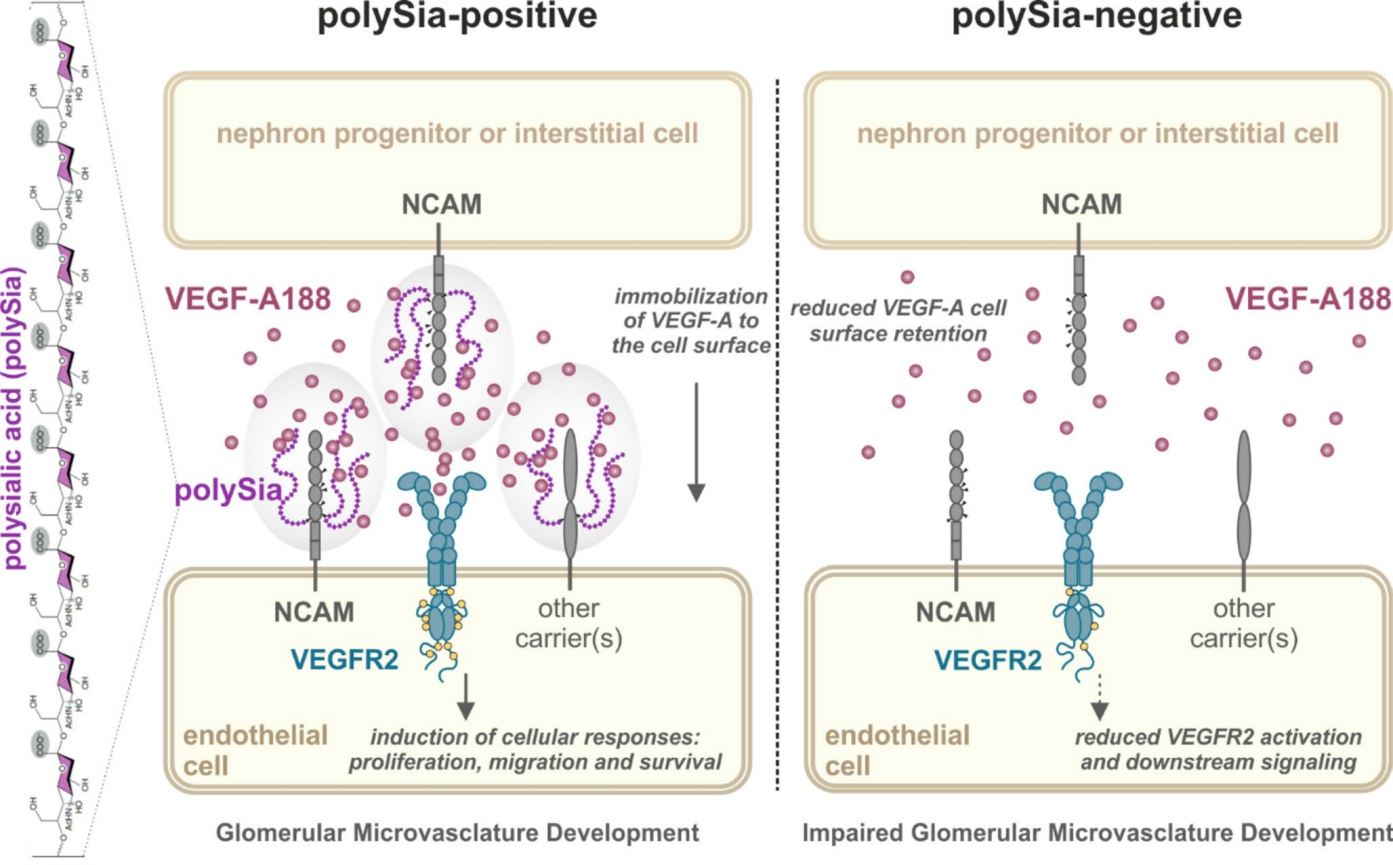

**Supplementary Information:**

The online version contains supplementary material available at 10.1007/s10456-025-09984-6.

## Introduction

Vascular endothelial growth factors (VEGFs) are key regulators of vasculogenesis and angiogenesis, two processes crucial in development and regeneration. The VEGF family comprises five members (VEGF-A, -B, -C, -D and placental growth factor (PlGF)), of which VEGF-A is the most potent angiogenic factor [1]. In early embryonic development VEGF-A deficiency is fatal in mice [2, 3]. In later stages, VEGF-A orchestrates the formation and functionality of the highly specialized glomerular vasculature [4]. Reduced VEGF-A expression in podocytes leads to a dysfunctional blood filtration barrier in the glomerular tuft and kidney failure due to impaired migration and proliferation of renal endothelial cells [5]. VEGF-A dysregulation is also associated with a variety of primary and acquired nephropathies [4, 6, 7] and plays a central role in the pathogenesis of diverse cancer types [8]. Thus, research on VEGF-A as potential therapeutic target for both anti- and pro-angiogenic therapies has gained momentum in the last decades [9, 10].

**Fig. 1:**
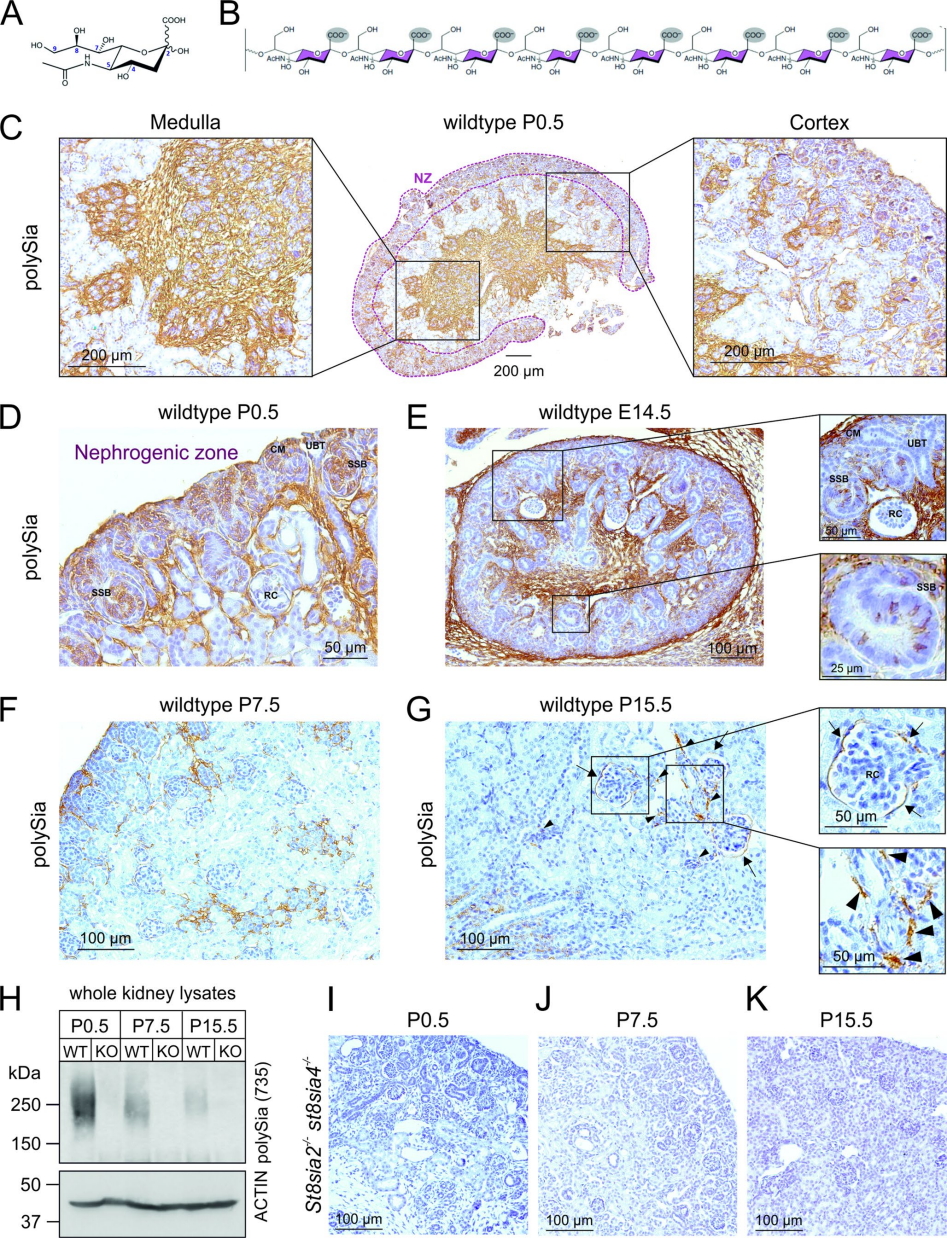
PolySia distribution in murine kidney. **(A)***N*-Acetylneuraminic acid. **(B)** PolySia DP8 scheme. **(C, D)** PolySia immunohistochemistry with 735 antibody on paraffin-embedded kidney sections from wildtype mice at postnatal day (P)0.5. **(C)** An entire stained renal section is shown to appreciate signal distribution with enlarged micrographs of the medullary and cortical area. Strong 735 staining is visible in the nephrogenic zone (NZ) and in the medulla. **(D)** Enlarged micrograph of the NZ, where different stages of nephron development are visible. **(E)** PolySia immunohistochemistry with 735 antibody on paraffin-embedded kidney sections from wildtype mice at embryonic day (E)14.5. An entire stained renal section is shown to appreciate signal distribution with enlarged micrographs of different stages of nephron development. **(F)** 735 immunohistochemistry of renal sections from P7.5 and **(G)** P15.5 old wildtype mice. PolySia expression decreases over time and residual cortical polySia staining at P15.5 is restricted to parietal epithelial cells (Bowman capsule, arrows) and cell layer adjacent to blood vessels (arrowheads). **(H)** Western blot analysis of kidney homogenates from wildtype (WT) and *St8sia2*^*−/−*^* St8sia4*^*−/−*^ (KO) mice at different postnatal time points. Protein concentrations of the samples were determined and 25 µg were loaded per lane. 735 staining shows decreasing signals in wildtype kidney over time and completely abolished signal in *St8sia2*^*−/−*^* St8sia4*^*−/−*^ kidneys at shown time points. Actin staining served as loading control. **(I)** 735 immunohistochemistry on renal sections of *St8sia2*^*−/−*^* St8sia4*^*−/−*^ mice at P0.5, **(J)** P7.5 and **(K)** P15.5. Representative micrographs of the cortical part are shown. CM, cap mesenchyme; RC, renal corpuscle (RC); SSB, S-shaped body; UBT, ureteric bud tip

While VEGF receptors (VEGFRs) 1 and 2 are both required for development and angiogenesis [11, 12], VEGFR2 is considered as the primary mediator of pro-angiogenic VEGF-A function. VEGF-A binding triggers VEGFR2 receptor dimerization, auto-phosphorylation and downstream signalling inducing cell migration, proliferation, cell survival and differentiation [13]. In mice, the three major pro-angiogenic isoforms VEGF-A120, -A164 and -A188 exist, corresponding to human VEGF-A121, -A165 and -A189, respectively. These isoforms differ in the extent of a C-terminal basic extension, which interacts with heparan sulfate proteoglycans (HSPGs) and thus regulates the immobilization of VEGF-A isoforms at the cell surface and in the extracellular matrix [14]. A recent study described the interaction of the glycocalyx component polysialic acid (polySia) with VEGF-A in vitro [15]. Polysialic acid, a linear homopolymer of α-(2 → 8)-linked 5-Acetamido-3,5-dideoxy-D-*glycero*-D-*galacto*-non-2-ulopyranosonic acid (*N*-Acetylneuraminic acid, sialic acid, Fig. 1A) monosaccharides (Fig. 1B), is a posttranslational modification of a small number of proteins and is part of the cell surface glycocalyx. The neural cell adhesion molecule (NCAM) is the most widely expressed and best studied polySia carrier [16]. The degree of polymerization (DP) of polySia chains varies between eight and DP > 90 monomers. Two Golgi-resident polysialyltransferases ST8SIA2 and ST8SIA4, enzymes with overlapping but distinct spatiotemporal expression patterns, are responsible for polySia biosynthesis [17]. Well-known to be essential for brain development and synaptic plasticity [18], polySia is also present in other organs during embryonic development, including liver [19], kidney [20, 21], heart [22] and testis [23]. Simultaneous deletion of both polysialyltransferases abolishes polySia synthesis and *St8sia2/St8sia4* double knockout mice develop major neurodevelopmental defects and die within four weeks after birth [24]. PolySia functions have mainly been ascribed to steric repulsion and masking effects. Beyond that, more recently, polySia has been identified as binding partner for specific signalling molecules, including fibroblast growth factor 2 (bFGF/FGF2) [25], brain-derived neurotrophic factor (BDNF) [26] and the chemokine CCL21 [27]. With the aim to identify in vivo a biological function for the recently described interaction of polySia with VEGF-A in vitro [15], we investigated postnatal renal development in a polySia-deficient mouse model, since balanced VEGF-A availability is critical and polySia expression has been reported during this period of renal development.

## Methods

### Mice and genotyping

The C57BL/6J mouse strain (C57BL/6JHanZtmBwei) served as control group in all experiments (RRID: IMSR_JAX:000664). Single animals were considered as experimental unit and allocated randomised. *St8sia2*^*−/−*^ (B6.129-St8sia2^tm1Jxm/J^, RRID:MGI:3699267) [28], *St8sia4*^*−/−*^ (B6.129Ola-St8sia4^tm1Crem^, RRID:MGI:3582,290) [29], *St8sia2*^*−/−*^* St8sia4*^*−/−*^ (B6.129-St8sia4^tm1Crem^-St8sia2^tm1Jxm^) mice [24] and *Ncam*^*−/−*^ (B6.129-Ncam1^tm1Cgn^) mice [30] (F6 backcross on C57BL/6JHanZtmBwei) were maintained in the animal facility of the Hannover Medical School under specific pathogen-free conditions in the same room according to ETS123 and 2010/63/EU. No sex differences were reported within the strains and only mice until P15.5 before onset of puberty were analysed. Genotypes were determined by PCR analysis as previously described [30]. Primers are listed in Supplemental Table S1.

### Ethical statement

All animal experiments were carried out in compliance with German law for protection of animals and were approved by the local authorities (*Niedersächsisches Landesamt für Verbraucherschutz und Lebensmittelsicherheit*, permission no. 2014/71 (2014–2019), 2019/244 (since 01.10.2019), 33.14–42,502-04_13_1312 (2013–2018), 33.12–42502-04-18_2932 (2018–2023), and AZ 33.12–42502-04–20/3510 (2020–2025); AZ 2019/244).

### Histology

Kidneys were fixed in 4% PFA/PBS and embedded in paraffin. Three µm sections were cut with a Leica RM2265 microtome (Leica Biosystems) and stained with Hematoxylin & Eosin (H&E) following standard procedures.

### Immunohistochemistry and indirect immunofluorescence

Detailed protocols are listed in Supplemental Methods.

### Urine analysis

Creatinine and total protein was determined in murine urine in the Olympus AU 400 analyser as described [31].

### Enzyme-linked immunosorbent assay

Plates (Greiner #675,101) were coated with purified *E.coli* K1 capsular polysaccharide composed α2,8-linked polySia of average DP 50 (avDP50) [32] and serial dilutions of recombinant murine VEGF-A isoforms 120, 164 and 188 (Reliatech, M30-032, M30-004, M30-094) were applied. Bound VEGF-A was detected with a pan-VEGF-A antibody, a HRP-conjugated secondary antibody (Supplemental Table S2) and 3,3’,5,5’-tetramethylbenzidine (Sigma) colour reaction. Absorbance was measured at 450 nm (Biotek) and analysis was performed with GraphPad Prism 5. For details see Supplemental methods.

### Horizontal native PAGE

200 pmol His-tagged VEGF-A188 was incubated in the presence of a fourfold molar excess of ligand in CSF buffer (1 mM sodium phosphate buffer containing 148 mM NaCl, 3 mM KCl, 1.4 mM CaCl_2_, 0.8 mM MgCl_2_ and 25 mM CaCl_2_) for 1 h at RT. The following ligands were used: (i) α2,8-linked polySia (avDP50 or DP24-30, generated as described in Schröder LJ et al. [33]) (ii) HS (Iduron) and (iii) Sia (Molekula Group), α2,8-linked di-Sia (Nakalai Tesque) and a tetramer of α2,8-linked Sia (Nakalai Tesque). Complexes were analysed in a horizontal 10% polyacrylamide gels. Electrophoreses was carried out in HEPES/Imidazol buffer (43 mM Imidazol, 35 mM HEPES, pH 7.4) or TBE buffer, pH 8.1 at 4 °C (according to McLellan [34]). Proteins were visualized by Coomassie brilliant blue staining. Histone from calf thymus (Sigma-Aldrich) and bovine serum albumin (BSA, Thermo Scientific™) were used as control proteins.

### Microscale thermophoresis

The interaction of VEGF-A188 with polySia (DP ≥ 40) and heparan sulfate (HS, Iduron GAG-HS01) was assessed by microscale thermophoresis (MST) as outlined in Wienken et al. [35]. The polySia pool was generated as described in Schröder LJ et al. [33] with the following modifications: Colominic acid sodium salt (Sigma Aldrich, #C5762) was subjected to limited hydrolysis in 100 mM acetic acid for 3 min at 70 °C. Subsequently, pH was adjusted to 10.5 with NaOH on ice, followed by 48 h incubation at 4 °C. The resulting mixture was processed at a concentration of 10 mg/mL through a 10 kDa cut-off filtering unit (Merck) to concentrate DPs of interest in the reservoir fivefold. Next, the upper fraction was diluted tenfold in loading buffer (10 mM Tris–HCl pH 8.0) and 3 mL aliquots were subjected to anion exchange chromatography. Fractionation was started at the conductivity equivalent of 478 mM NaCl (elution buffer: 1 M NaCl with 10 mM Tris–HCl pH 8.0) and pooled isolated fractions were desalted applying a 3 kDa cut-off filtering unit (Merck). Recombinant VEGF-A188-V5-His was fluorescently labelled with Alexa Fluor™ 647 NHS Ester (Invitrogen™) and purified using Slide-A-Lyzer™ 3,5 K MWCO MINI (Thermo Fisher Scientific). Achieved degree of labeling was 0.5 (fluorophor:protein). Labelled VEGF-A at a final concentration of 10 nM was incubated with increasing concentrations of polySia DP ≥ 40 (391 nM to 400 µM) or HS (16.26 nM to 16.6 µM) in assay buffer (20 mM HEPES pH 8.0, 150 mM NaCl) at 30 °C for 30 min, shaking. MST was performed in premium glass capillaries (NanoTemper Technologies) on a Monolith NT.115 Pico device (NanoTemper Technologies). Thermophoresis was induced with infrared-laser power set to 40% and fluorescence was induced with the red LED channel at 20% excitation power. Normalized thermophoresis fluorescence averaged from 3 to 5 experiments was plotted against ligand concentration and K_D_ values were calculated based on the Hill equation (Origin 2020b software, OriginLab Corporation).

### Vegfa188 BaseScope assay

BaseScope assay was performed on 5 µm paraffin-embedded kidneys sections of newborn C57Bl/6J wildtype mice according to the manufacturers protocol (Advanced Cell Diagnostics). The BaseScope probe BA-Mm-Vegfa-E6-1zz-st (ACD # 821111) was used to detect *Vegfa* transcript variant 1 mRNA (VEGF-A188, NM_001287056.1) with the BaseScope detection reagent kit v2 RED (ACD # 323900).

## Kidney spectral flow cytometry

Kidneys of mice at the age of P0.5 and P3.5 were minced and incubated with 1 mg/mL of Collagenase IV (Invitrogen) in RPMI for 1 h at 400 rpm, and filtered through a 70 µm mesh. Cells were pelleted, washed with cold PBS and stained with Zombie NIR (1:1000 in PBS, Biolegend) on ice for 20 min. Cells were washed with PBS and incubated with Fc block (1:2000 in PBS-2%FCS, Biolegend) on ice for 10 min. Incubation with antibodies and in house purified biotinylated inactive Endosialidase (iEndo) [36] and subsequently with secondary antibodies was performed in PBS-2%FCS on ice for 30 min. Samples were measured on a Aurora spectral flow cytometer (Cytek Biosciences) with autofluorescence extraction. Compensation beads (Thermo Fisher) or cells were used for single-stained controls. Data was analysed with FlowJo 10.1.

### Single-cell RNA sequencing data processing

Publicly available single-cell RNA sequencing datasets of embryonic day 15 (E15) and P0 murine kidneys (GEO accession number GSM4648414) [37] and from human fetal kidney at post-conception weeks 9–12 [38] were downloaded and further processed as described in Supplemental methods and Supplemental file 3.

### VEGFR2 activation

Near confluent HUVECs were starved overnight in supplemented EGM-2 cell culture medium (Lonza) lacking VEGF. Cells were treated with 1 µg/mL endosialidase F (Endo) in PBS for 30 min at 37 °C, washed with PBS and stimulated with 25 ng/mL recombinant VEGF-A188-V5-His for 5 min at 37 °C in EGM-2 supplemented with 0.5% FBS. After washing with ice-cold PBS, cells were lysed in RIPA buffer and submitted to SDS-PAGE and western blot analysis. For details see Supplemental methods.

### Statistics

Due to the small samples sizes non-normal distributions were assumed and non-parametric statistical tests were applied. Statistical analyses were performed with Mann–Whitney test for comparison of two groups, and Kruskal–Wallis Test with Dunn’s multiple comparison for comparison of more than two groups (GraphPad PRISM 9). Data points are depicted as individual values with mean and SD. At least three technical or biological replicates were included in each experiment and the analysed replicate number (n) is indicated.

## Results

### PolySia is expressed during embryonic and postnatal murine kidney development

To explore possible implications of polySia in the glomerular vasculature development, we examined the spatiotemporal polySia expression during postnatal kidney development in wildtype mice. We performed immunohistochemical stainings with the monoclonal antibody (mAb) 735, detecting polySia with DP ≥ 10 [39, 40] (Fig. 1C). Specific binding was confirmed routinely by the treatment of consecutive tissue sections with endosialidase F (Endo), specifically degrading polySia [41] (Supplemental Fig. S1). At birth, the murine kidney exhibited robust polysialylation with prominent staining in the cortical area including the nephrogenic zone (NZ), where nephrogenesis occurs, and the medulla (Fig. 1C). At post natal day (P)0.5, the formation of new nephrons, the functional units of the kidney, is still in progress and polySia is found on interstitial cells and different stages of nephron development, like the cap mesenchyme (CM), comma-shaped bodies (CB) and S-shaped bodies (SSB). The renal corpuscle (RC) which forms the blood-filtering unit of the mature nephron, tubular structures and ureteric bud tips (UBTs) that give rise to the collecting duct system were devoid of polySia (Fig. 1D). A similar pattern was observed in renal sections at embryonic day (E)14.5 (Fig. 1E), with polySia expression in interstitial cells as well as CM and SSBs, but not in RCs and UBTs. As renal development progresses for at least one week after birth, we monitored polySia expression at P7.5 and P15.5. Although decreasing with age, polySia expression continued (Fig. 1F, G). By the time of kidney maturation (P15.5), its expression became confined to regions adjacent to the Bowman capsule, interstitial cells adjoining blood vessels and tubules, and medullary interstitial cells. Western blot analysis of wildtype kidney homogenates confirmed the decrease of polySia expression during postnatal development (Fig. 1H). We neither observed polySia in kidney homogenates nor at cellular level in renal sections from *St8sia2*^*−/−*^* St8sia4*^*−/−*^ mice (Fig. 1I-K). Collectively, these findings suggest a pivotal role for this glycan with regulated spatiotemporal expression pattern in kidney morphogenesis.

### PolySia-deficient mice display reduced numbers of glomerular endothelial cells

**Fig. 2:**
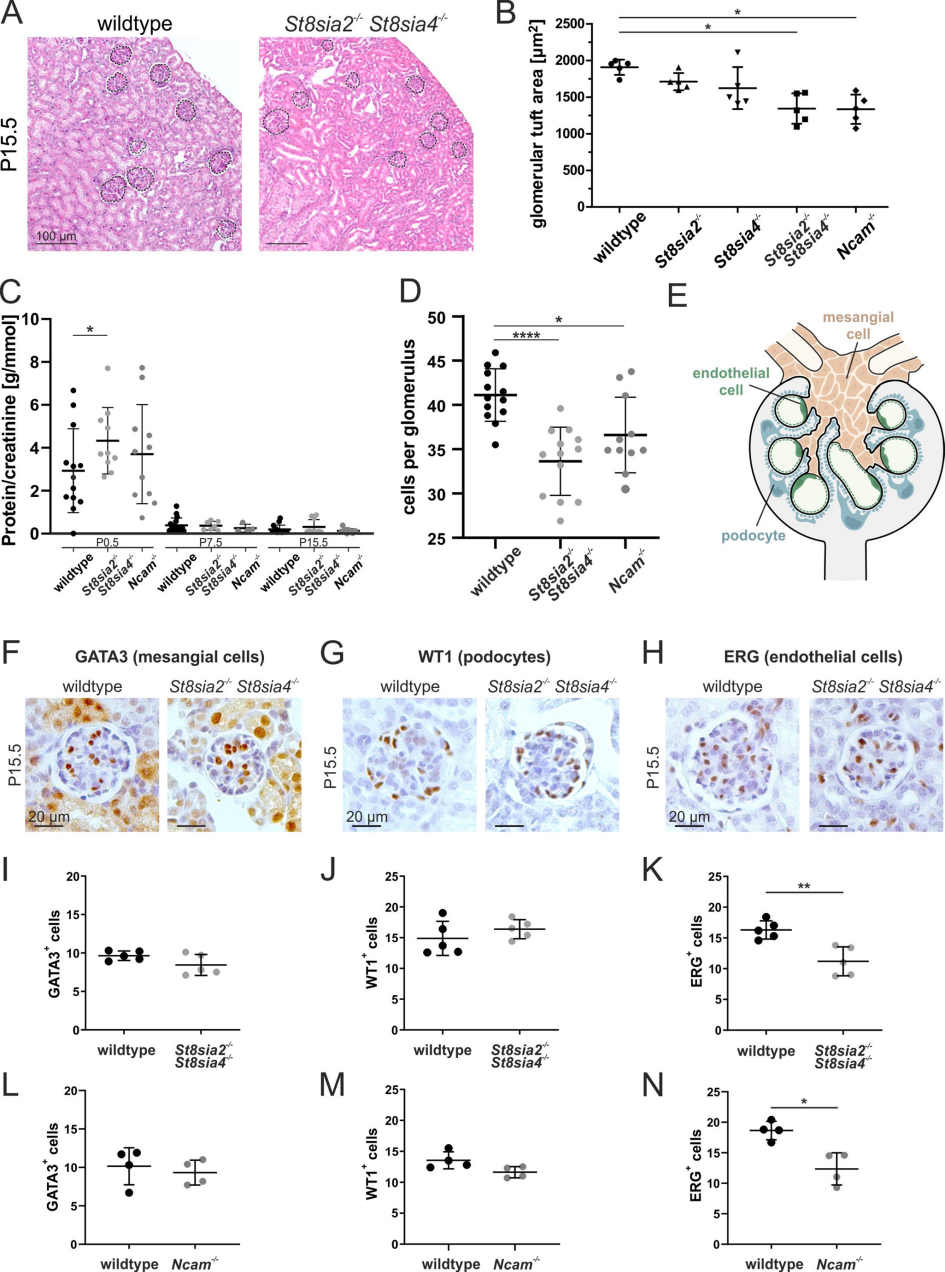
Analysis of polySia-deficient murine kidney. **(A)** H&E staining of paraffin-embedded kidney sections from wildtype and *St8sia2*^*−/−*^* St8sia4*^*−/−*^ mice (P15.5). Glomeruli in the renal cortex are framed. **(B)** Glomerular tuft areas measured in H&E stained kidney sections (P15.5) of n = 5 wildtype and mutant mice, respectively. Per section, all visible glomerular tufts were measured. Means ± SD and individual data points are depicted. Non-parametrical Kruskal–Wallis test (p = 0.0032) and Dunn’s multiple comparison indicated significant group differences (* p < 0.05). **(C)** Protein/creatinine ratios measured in urine from wildtype, *St8sia2*^*−/−*^* St8sia4*^*−/−*^ and *Ncam*^−/−^ mice collected at different postnatal time points (P0.5, 7.5 and 15.5). Means ± SD and individual data points of n = 4–16 individuals are depicted. Nonparametric Mann–Whitney test indicated significant differences between wildtype and *St8sia2*^*−/−*^* St8sia4*^*−/−*^ mice at P0.5, during the maturation of the blood filtration barrier (* p < 0.05, highlighted). For clarity reasons, significant differences during kidney maturation, between P0.5-P7.5 and P0.5-P15.5, for all three indicated genotypes are not indicated. No significant differences were detected between the genotypes for the time points P7.5 and P15.5. **(D)** Total cell numbers per glomerulus were assessed on kidney sections from wildtype, *St8sia2*^*−/−*^* St8sia4*^*−/−*^ and *Ncam*^−/−^ mice (P15.5) by counting hematoxylin stained nuclei and WT-1, ERG, or GATA3 immunoreactive cells (see F, G and H) or hematoxylin-stained cell nuclei in H&E stained renal sections. N = 13 wildtype, N = 13 *St8sia2*^*−/−*^* St8sia4*^*−/−*^ animals and N = 11 *Ncam*^*−/−*^ animals and per section 30 glomeruli in comparable areas of the tissues were analysed. Means ± SD and individual data points are depicted. Statistical significance was tested with a nonparametric Mann–Whitney-U test (* p < 0.05; **** p < 0.0001). **(E)** Scheme of a mature glomerulus composed of three different cell types in the glomerular tuft: podocytes (blue), endothelial cells (green) and mesangial cells (orange). **(F–H)** Immunohistological staining of nuclear localized glomerular cell type markers on paraffin-embedded kidney sections (wildtype and *St8sia2*^*−/−*^* St8sia4*^*−/−*^, P15.5): GATA3 staining for mesangial cells **(F)**, WT1 staining for podocytes **(G)** and ERG staining for endothelial cells **(H)**. Evaluation of cell numbers of **(I)** GATA3^+^ cells, **(J)** WT1^+^ cells and **(K)** ERG^+^ cells (** p < 0.01, nonparametric Mann–Whitney test) from immunohistological staining on paraffin-embedded kidney sections (P15.5) of wildtype and *St8sia2*^*−/−*^* St8sia4*^*−/−*^ mice. **(L-N)** Analysis of renal sections from *Ncam*^−/−^ and wildtype mice at P15.5 regarding the evaluation of cell numbers of **(L)** GATA3^+^ cells, **(M)** WT1^+^ cells and **(N)** ERG^+^ cells (* p < 0.05, nonparametric Mann–Whitney test). n = 4–5 individuals were analysed per genotype and per section 30 glomeruli were selected for counting in equal areas of the section. Data is displayed as averages per mouse with means and standard deviation for the group

Next, we comparatively analysed kidneys isolated from wildtype and *St8sia2*^*−/−*^* St8sia4*^*−/−*^ mice. Mutant kidneys were similar in gross morphology to wildtype kidneys, and the overall kidney architecture appeared unaltered, as evidenced by in situ hybridization (Supplemental Fig. S2). In H&E stained kidney sections of mature kidneys (P15.5), the glomeruli of polySia-negative mice appeared smaller compared to control animals (Fig. 2A), with morphometric analysis confirming significantly smaller glomerular tuft areas in *St8sia2*^*−/−*^* St8sia4*^*−/−*^ mice (Fig. 2B). Notably, a similar histological phenotype was seen in mice that are deficient in NCAM, the major polySia carrier protein [42], but not in single knockout mice lacking either *St8sia2*^*−/−*^ or *St8sia4*^*−/−*^ (Fig. 2B). Next, we analysed the functionality of blood filtration by measuring protein/creatinine ratios in urine samples from control and polySia-negative mice from P0.5—P15.5 (Fig. 2C). Consistent with the immature blood filtration barrier in newborn mice, low-level proteinuria was apparent in both genotypes. While proteinuria, given by protein/creatinine ratio, was increased in *St8sia2*^−/−^*St8sia4*^−/−^ mice at P0.5, we did not observe genotype-specific proteinuria at later time points, indicating that loss of polySia did not affect the mature glomerular filtration barrier. By quantifying glomerular cell numbers, in mature kidneys (P15.5), we observed significantly reduced total glomerular cell counts in *St8sia2*^*−/−*^* St8sia4*^*−/−*^ animals as compared to control mice (Fig. 2D). Staining of the three glomerular cell types (Fig. 2E) with cell type-specific nuclear markers revealed unaltered numbers of GATA3-positive mesangial cells (Fig. 2F, I) and of WT1-positive podocytes (Fig. 2G, J). In contrast, ERG-positive glomerular endothelial cells were reduced in *St8sia2*^*−/−*^* St8sia4*^*−/−*^ mice by about 30% (Fig. 2H, K). As mentioned, on renal sections of *Ncam*^−/−^ mice at P15.5, we observed reduced glomerular tuft areas (Fig. 2B), but overtly normal kidney function (Fig. 2C). Numbers of total glomerular cells (Fig. 2D) and glomerular endothelial cells were reduced in *Ncam*^−/−^ mice compared to control mice (Fig. 2L-N). Thus, *Ncam*^−/−^ mice resembled the phenotype of the *St8sia2*^*−/−*^* St8sia4*^*−/−*^ mouse model. These results point towards a role of polySia presentation on NCAM for nephron development, and specifically for the formation of the glomerular microvasculature.

### PolySia interacts with VEGF-A188 in vitro

**Fig. 3:**
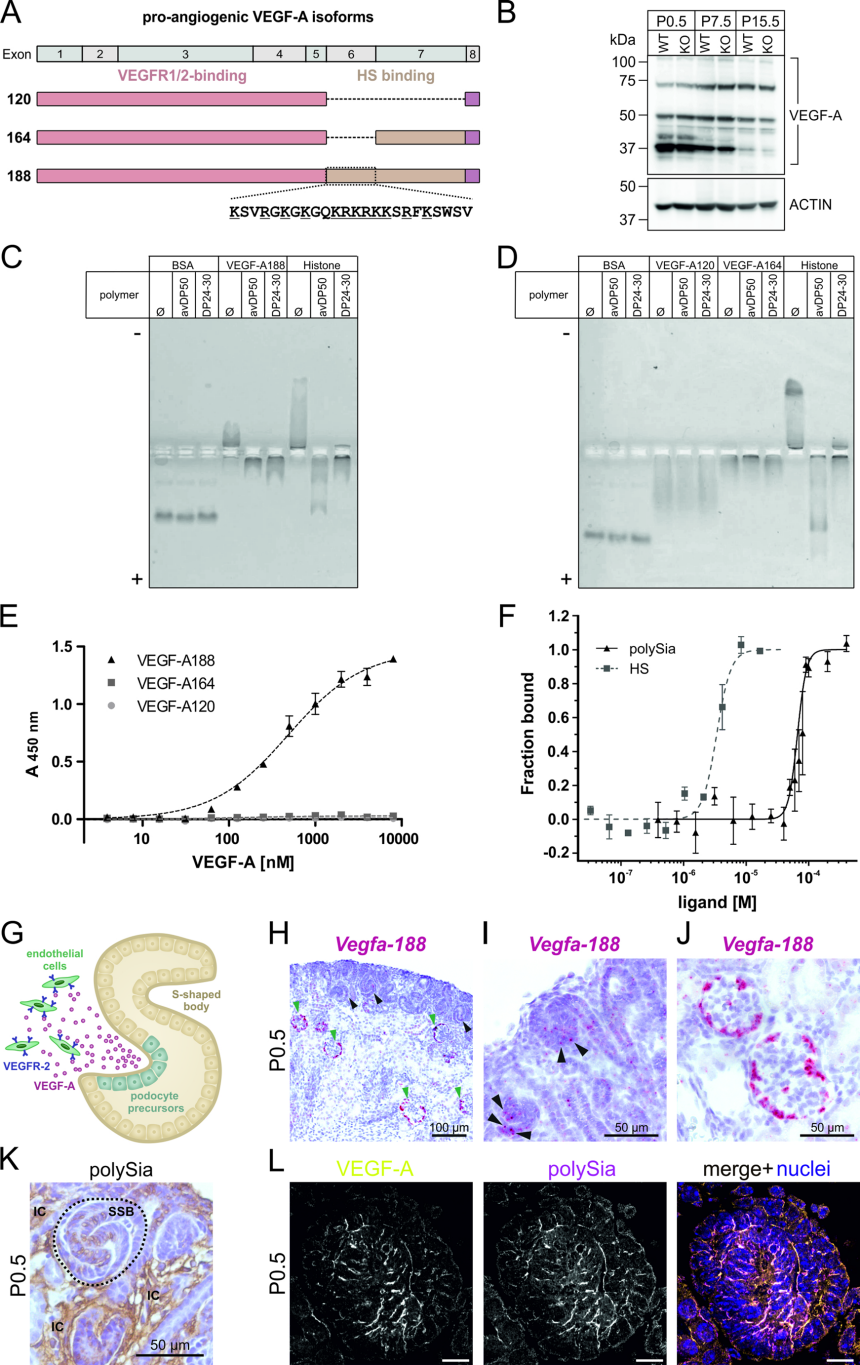
VEGF-A isoform expression in murine kidney, interaction with polySia in vitro and spatial relationship between VEGFA and polySia during nephron development **(A)** Scheme of the exon structure and the three major murine pro-angiogenic VEGF-A isoforms 120, 164 and 188. VEGFR and heparan sulfate (HS) binding sites are indicated. 24 amino acid sequence of exon 6, which is only present in VEGF-A188 is displayed and basic residues are underlined. **(B)** Western blot analysis of wildtype (WT) and *St8sia2*^*−/−*^* St8sia4*^*−/−*^ (KO) kidney homogenates prepared at different postnatal time points. Protein concentrations of the samples were determined and 25 µg were loaded per lane. Staining with a VEGF-A antibody results in multiple bands. Actin staining serves as loading control. **(C, D)** Binding analysis of **(C)** VEGF-A188 and **(D)** VEGF-A120 and -A164 with polySia of different chain length (average DP50 (avDP50) and DP24-30) in horizontal native PAGE. Bovine serum albumin (BSA) is shown as a negative control and histone as a known polySia interaction partner is shown as a positive control. Complex formation of histone with both polySia pools increased the negative charge of the protein reflected by a reversion of the migration direction in the native PAGE (pH = 8.1). **(C)** Complex formation of VEGF-A188 (pI = 9.25) with avDP50 and DP24-30 reverted the migration direction towards the anode ( +) in horizontal native PAGE (pH = 7.4), as observed for histone. **(D)** In contrast, the mobility of BSA (pI ~ 5) and the two VEGF isoforms -A120 (pI = 6.48) and -A164 (pI = 7.93) was not grossly affected by either long chain (avDP50) or shorter chain polySia (DP24-30). Both polySia pools did not increase the covered migration distance of the VEGF-A isoforms to the anode, indicating a lack of interaction with polySia. Native PAGE conditions allowing optimal migration in PAGE were used for all three proteins. ( +) anode, (-) cathode. **(E)** ELISA of VEGF-A isoforms binding to polySia. PolySia of avDP50, derived from *E. coli* K1 capsule polysaccharide, was immobilized on a plate. Mean and standard deviation calculated from three individual experiments are shown with horizontal axis in log scale. The dotted lines show a non-linear fit to the mean values of the individual isoforms. Binding is observed only for the VEGF-A isoform 188. **(F)** Protein-glycan interaction analysis by microscale thermophoresis. Binding curves illustrate the interaction of fluorescently labelled VEGF-A188 with polySia (DP ≥ 40) and heparan sulfate (HS) as positive control. Normalized thermophoresis fluorescence averaged from 3 to 5 independent experiments is plotted against ligand concentration and K_D_ values were calculated based on the Hill equation (K_D_(polySia) = 66.1 ± 3.17 µM, K_D_(HS) = 3.38 ± 0.77 µM). The error bars report the standard deviation. **(G)** Illustration of endothelial (precursor) cells migration into the S-shaped body of a developing nephron. Podocyte precursors (blue) secrete VEGF-A (red) and thereby attract VEGFR-2 (dark purple) expressing endothelial cells (green) into the vascular cleft of the S-shaped body. **(H)***Vegfa188* base scope assay was performed on paraffin-embedded kidney sections from newborn wildtype mice. *Vegfa188* mRNA is expressed in developing (black arrowheads) and mature (green arrowheads) glomeruli in the renal cortex. Micrographs with higher magnification clearly show intense red staining in **(I)** developing nephrons (black arrowheads) and **(J)** mature glomeruli, in which the outer cell layer (podocytes) is intensively stained. **(K)** Immunohistochemical staining for polySia on renal section (wildtype P0.5) with 735 antibody. Intense polySia staining is visible in the centre of an S-shaped body (circled with dashed line). **(L)** Immunofluorescence co-staining for polySia and VEGF-A on murine kidney section (wildtype P0.5). PolySia (magenta) and VEGF-A (yellow) staining is visible in the developing glomerulus (S-shaped body) and overlapping signals are shown in white in the merged image. Cell nuclei are stained with DAPI (blue)

The reduced endothelial cell number in glomeruli of polySia-deficient mice resembles the compromised development of the glomerular microvasculature in mouse models with impaired VEGF-A expression [5, 43]. However, immunoblotting revealed that all three pro-angiogenic isoforms VEGF-A120, -A164 and -A188 (Fig. 3A), were present at similar levels in kidney homogenates from wildtype and *St8sia2*^*−/−*^* St8sia4*^*−/−*^ mice (Fig. 3B). This observation was corroborated by qPCR analysis, showing similar mRNA levels of all three isoforms as well as the corresponding receptor VEGFR2 (Supplemental Fig. S3). Since VEGF-A and VEGFR2 at the gene expression and protein level was not altered by polySia-deficiency, we hypothesized that the interaction of polySia with VEGF-A itself modulates VEGF-A signalling and regulates glomerular endothelial cell numbers. To investigate the binding of VEGF-A to polySia in vitro, we incubated all three VEGF-A isoforms with purified polySia and analysed the formation of protein-polysaccharide complexes by horizontal native PAGE (Fig. 3C, D). Specifically, we used size-fractionated polySia with a degree of polymerization (DP) of 24–30 [33], which reflects the most abundant DP of polySia on perinatal brain NCAM [44, 45], and polySia with an average DP of 50 (avDP50). In the given experimental setup, binding to the polyanionic polysaccharide polySia is indicated by an increased electrophoretic mobility of the bound protein towards the anode ( +). In Fig. 3C and D, this is exemplified for the known polySia-histone interaction [46], which served as a positive control. Bovine serum albumin (BSA) was used as a non-binding control protein. Notably, VEGF-188 showed an electrophoretic mobility similar to histones. While VEGF-188 alone migrated towards the cathode, it showed increased mobility towards the anode when pre-incubated with polySia of avDP50 or DP24-30 (Fig. 3C), indicating the formation of a polySia-VEGF-A188 complex. For VEGF-A120 and -A164, however, we observed no significant change in electrophoretic mobility upon pre-incubation with polySia (Fig. 3D). The suitability of our horizontal native PAGE approach for monitoring protein–polysaccharide interactions [25], was further confirmed with heparan sulfate (HS), a polysaccharide that is known to sequester VEGF-A188 and -A164, but not VEGF–A120, to the cell surface [14]. In the presence of HS, VEGF-A188 showed an increased mobility to the anode (Supplemental Fig. S4A), which was seen to a lesser extend for VEGF–A164, but not for VEGF-A120 (Supplemental Fig. S4B). Importantly, the electrophoretic mobility of VEGF-A isoforms was not affected by the addition of mono-Sia, α2,8-linked di-Sia or oligo-Sia with DP4 (Supplemental Fig. S4C, D). When we analysed VEGF-A binding in a photometric ELISA with immobilized bacterial polySia of avDP50 as shown schematically in Supplemental Fig. S5, we observed binding for VEGF-A188, but not for VEGF-A164 or -A120 (Fig. 3E). This result confirmed an isoform-specific binding and indicates that polySia-VEGF-A interaction can be attributed to the VEGF-A188 specific 24 amino acids that are encoded by exon 6 (Fig. 3A). A comparative analysis of VEGF-A188 binding to polySia of DP > 40 and HS was performed by microscale thermophoresis (MST) (Fig. 3F). This method measures protein-glycan interactions by tracking the molecule movement within a temperature gradient. The dissociation constants (K_D_) calculated from the binding curves shown in Fig. 3F, revealed a 20-fold lower affinity for the polySia-VEGF-A188 binding (K_D_ 66.1 ± 3.17 µM) compared to the HS-VEGF-A188 binding (K_D_ 3.38 ± 0.78 µM). This newly identified interaction between polySia and VEGF-A188 provides a potential mechanism how this glycan promotes VEGF-A signalling and glomerular vasculature development.

### VEGF-A188 and polySia are concurrently expressed in the developing nephron

During nephron development, presumptive podocytes secrete VEGF-A to induce endothelial precursor cell migration into the vascular cleft of the developing nephron in the S-shaped body (Fig. 3G) to later form the glomerular tuft [47]. A crucial prerequisite for a possible interaction between VEGF-A188 and polySia in vivo is the spatiotemporal colocalization of the two molecules. Using the BaseScope in situ hybridization approach (Supplemental Fig. S6), we observed VEGF-A188 expression in both, comma- and S-shaped bodies of the developing nephron (Fig. 3H, I, black arrowheads) and additionally in mature glomeruli (Fig. 3H, J, green arrowheads). Immunohistological staining revealed the presence of polySia on cells of the S-shaped body as well as on interstitial cells (IC) surrounding the developing nephron (Fig. 3K). Co-staining with a pan-VEGF-A antibody and the anti-polySia antibody 735 confirmed the co-localization of both factors in developing nephrons of newborn wildtype kidney (Fig. 3L).

### Identification of polysialyltransferase-expressing cell types during kidney development

**Fig. 4:**
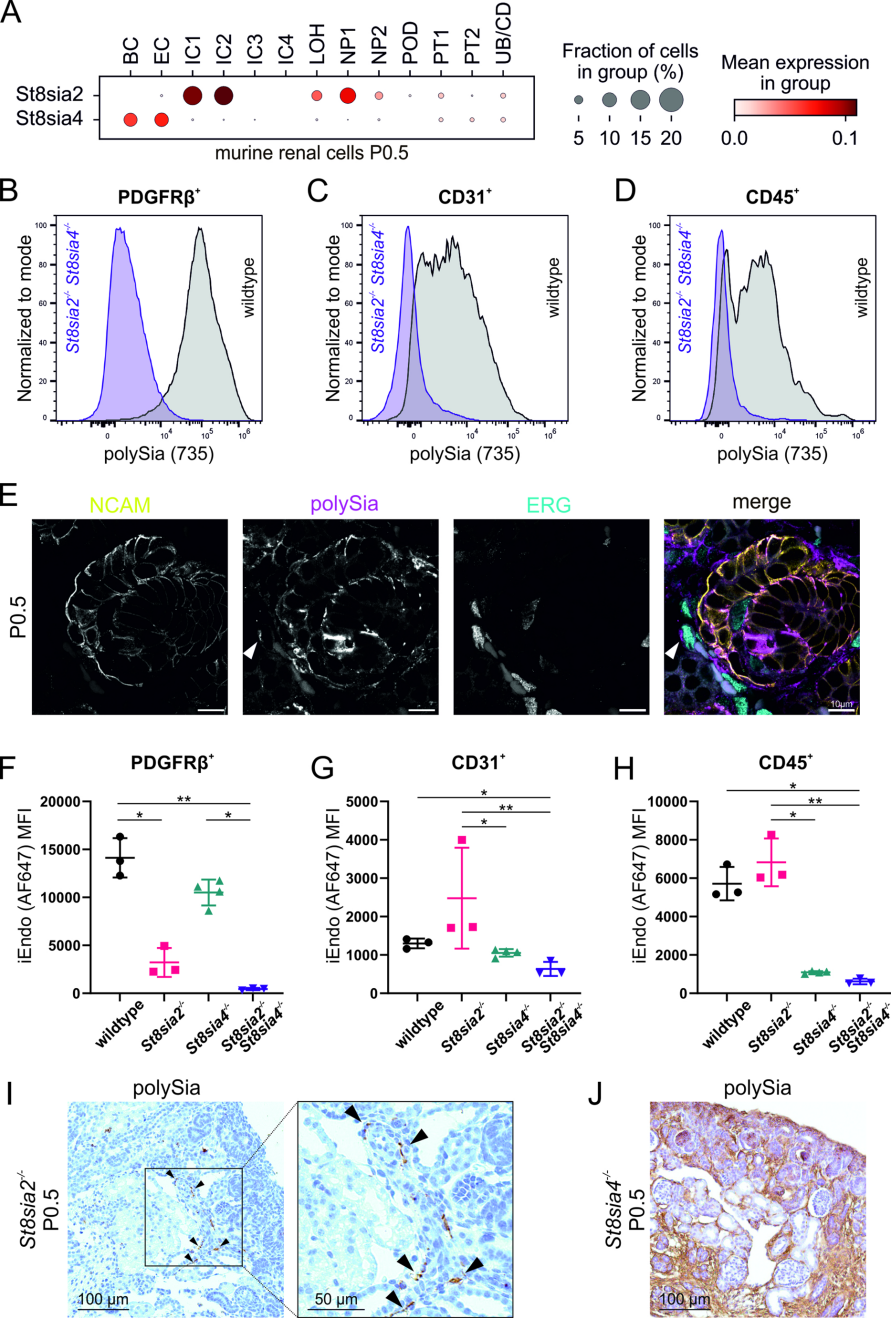
PolySia expression in distinct renal cell types and contribution of the polysialyltransferases ST2SIA2 and ST8SIA4 during postnatal renal development. **(A)** Evaluation of polysialyltransferase gene expression in different renal cell types (Leiden clusters) in P0 wildtype mice. Single cell RNAseq data (Accession no. GSM4648414) was obtained from Naganuma et al*.* (2021). LOH: loop of Henle, IC: interstitial cell, EC: endothelial cell, NP: nephron progenitor (describing cells of the cap mesenchyme and the nascent nephron), PT: proximal tubule, UB: ureteric bud tip, CD: collecting duct, POD: podocyte, BC: blood cell. **(B)** Normalized histograms of spectral flow cytometry analysis from kidney single cell suspensions of newborn wildtype and polySia-deficient (*St8sia2*^*−/−*^* St8sia4*^*−/−*^) mice. Cells were stained for polySia with the 735 antibody and renal cell type markers for interstitial cells (PDGFRβ), **(C)** endothelial cells (CD31) and **(D)** immune cells (CD45)**. (E)** Immunofluorescence co-staining of NCAM (yellow), polySia (magenta) and the endothelial cell marker ERG (cyan) on renal section of newborn wildtype mice. Partial colocalisation is observed in cells of an S-Shaped body. Overlapping signals are coloured white in the merged image. The white arrowhead indicates a polySia-positive endothelial cell. **(F–H)** Spectral flow cytometry analyses of renal single cell suspensions from newborn wildtype and polysialyltransferase knockout mouse strains *St8sia2*^*−/−*^*, St8sia4*^*−/−*^* and St8sia2*^*−/−*^* St8sia4*^*−/−*^*.* Cell suspensions were stained for polySia with inactive endosialidase (iEndo) and for interstitial cells (PDGFRβ, **F**), endothelial cells (CD31, **G**) and immune cells (CD45, **H**) to discriminate between different cell types**.** The calculated median fluorescence intensities (MFI) are shown with each data point representing one biological replicate (n = 4 for *St8sia4*^*−/−*^, n = 3 for others). Means and standard deviations are depicted. Non-parametrical Kruskal–Wallis test indicated significant differences (PDGFRβ, CD31: p < 0.0001, CD45: p = 0.0001). Uncorrected Dunn’s post hoc tests were applied and significant group differences are indicated (* p < 0.05, ** p < 0.01). Representative histograms for the results depicted in B-D are shown in Supplemental Figure S8G-I. **(I)** PolySia (735) staining on renal sections of newborn *St8sia2*^*−/−*^ and **(J)***St8sia4*^*−/−*^ mice. Cortical areas of the tissue are depicted to appreciate polySia staining in the nephrogenic zone. Residual polySia signal in *St8sia2*^*−/−*^ kidney on interstitial cells is marked with arrowheads

Publicly available neonatal kidney single-cell RNA sequencing data from C57BL/6N mice [37] was used to evaluate the cell types with putative polySia presence, by the analysis of *St8sia2* and *St8sia4* gene expression at P0.5 (Fig. 4A, Supplemental Fig. S7A, B). Expression of the two polysialyltransferase genes was detected in five selected cell types with a score above a threshold of > 5% of cells in the respective group. In line with polySia-positive cells of the S-shaped body detected in immunostained renal sections (Fig. 1C and E, Fig. 3K and L), approximately 10% of the nephron progenitor 1 (NP1) cluster displayed the expression of *St8sia2*. The NP clusters are built by *Six2*-positive nephron progenitor cells found in the cap mesenchyme and in all cells of the nascent nephron [48]. Immunofluorescent costaining of polySia with NCAM, which is found to be expressed in all stages of nephron development [49], we observed co-localization of polySia and NCAM on the surface of the cells in the cap mesenchyme as well as in comma- and S-shaped bodies of renal sections from P0.5 wildtype mice (Supplemental Fig. 7C). In contrast to *St8sia2*, *St8sia4* was present in about 10% of endothelial cells (EC) and blood cells (BC). Corresponding to the known heterogeneity of renal interstitial cells (IC) [50, 51] four IC clusters were identified, with *St8sia2* expression in about 13% of cells in the clusters IC1 and IC2 (Fig. 4A). Low levels of *St8sia2* expression were assigned to a subset of about 10% of cells of the loop of Henle in mature nephrons. Analysis of the respective scRNAseq data set from embryonic day 15 (E15) [37] revealed a similar gene expression pattern of the two polysialyltransferases *St8sia2* and *St8sia4* as seen at P0.5 (Supplemental Fig. S7D, E). In line with this, polySia was detected on renal sections from wildtype mice at E14.5, showing co-localization with NCAM on cells of S-shaped bodies (Supplemental Fig. S7F). Altogether, a highly cell type-specific expression of *St8sia2* and *St8sia4* was detected in the developing kidney of wildtype mice.

### PolySia is expressed on endothelial precursor cells in spatial proximity to developing nephrons

Polysialylation of the different renal cell types was further assessed by spectral flow cytometry from whole kidney cell isolation, in newborn wildtype mice. Samples from *St8sia2*^*−/−*^* St8sia4*^*−/−*^ mice were also analysed (Fig. 4B-D, gating strategy in Supplemental Fig. S8A-E). Platelet Derived Growth Factor Receptor beta (PDGFRβ^+^) was used as a cell surface marker for the heterogeneous group of renal interstitial cells including several types of interstitial fibroblasts, vascular smooth muscle cells, glomerular mesangial cells, parietal epithelial cells and pericytes. In line with strong polySia signals detected in the interstitium of renal sections (Fig. 1C and 3K), we observed a strong polySia signal on all cells of the PDGFRβ^+^ population (Fig. 4B). Endothelial cells (CD31^+^) were also identified as polySia-positive (Fig. 4C), but with a lower signal intensity compared to the PDGFRβ^+^ cell population. Finally, a subset of cells that were positive for the pan-leukocyte marker CD45 displayed polySia (Fig. 4D). PolySia signals were lost in *St8sia2*^*−/−*^* St8sia4*^*−/−*^ mice (Fig. 4B-D). The identification of polySia-positive endothelial cells led to the hypothesis that polysialylation fosters the VEGF-A guided migration of endothelial precursor cells into the vascular cleft of developing nephrons and/or promotes differentiation processes that are essential for the formation of the glomerular microvasculature. Using confocal immunofluorescence microscopy, we observed polySia on ERG-positive endothelial cells that are in close proximity to developing nephrons, as shown exemplarily for an S-shaped body at P0.5 (Fig. 4E) and at E14.5, exemplarily shown for a comma-shaped body and developing renal epithelium (Supplemental Fig. S8F). In line with this, the polysialyltransferase ST8SIA4 is expressed in endothelial cells in both developmental stages (Fig. 4A, Supplemental Fig. S7E). Moreover, we detected polySia in the vascular cleft of S-shaped bodies (Fig. 4E). The assignment to a specific cell type(s) however, is challenging due to the close proximity of polySia-positive cells of the developing nephron and infiltrating endothelial precursor cells. Together, our combined data set obtained by flow cytometry, histological stainings and the analysis of scRNAseq data demonstrated polySia in interstitial cells, as well as subtypes of nephron progenitors and endothelial cells in the developing kidney and illustrate how cell-type specific expression of *St8sia2* and *St8sia4* determine the polySia pattern in the developing kidney.

### Both polysialyltransferases, ST8SIA2 and ST8SIA4, contribute to polysialylation during nephron development

Based on the finding that the two polysialyltransferases showed only partially overlapping expression profiles (Fig. 4A), we assessed the individual contribution of ST8SIA2 and ST8SIA4 to polysialylation in selected renal cell types by spectral flow cytometry. As expected, polysialylation was abolished in all analysed renal cell types of newborn *St8sia2*^*−/−*^* St8sia4*^*−/−*^ mice (Fig. 4F-H, representative histograms are shown in Supplemental Fig. S8G-I). In accordance with the scRNAseq data, the major portion of polySia in PDGFRβ^+^ renal interstitial cells was synthesized by ST8SIA2 (Fig. 4F). In comparison to interstitial cells, we observed considerably lower polysialylation levels in CD31^+^ endothelial cells in wildtype kidney, but apparently, both polysialyltransferases were able to contribute to endothelial cell polysialylation (Fig. 4G). However, compensatory mechanisms in mice lacking only one polysialyltransferase cannot be excluded. PolySia levels in CD45^+^ leukocytes of *St8sia4*^*−/−*^ kidneys were reduced to the level in polySia-negative mice, validating ST8SIA4 as the enzyme responsible for polysialylation in immune cells (Fig. 4H). Complementary to the spectral flow cytometry analysis we conducted immunohistological staining for polySia on kidney sections of newborn *St8sia2*^*−/−*^ and *St8sia4*^*−/−*^ mice. The polySia signal in *St8sia2*^*−/−*^ mice was strongly reduced with residual staining found on a small fraction of cells in the interstitium (Fig. 4I, arrowheads). In *St8sia4*^*−/−*^ renal sections, the polySia signal was comparable to wildtype levels with strong signal in the cortical region especially in the interstitium (Fig. 4J).

### NCAM is the major polySia carrier in murine kidney

**Fig. 5:**
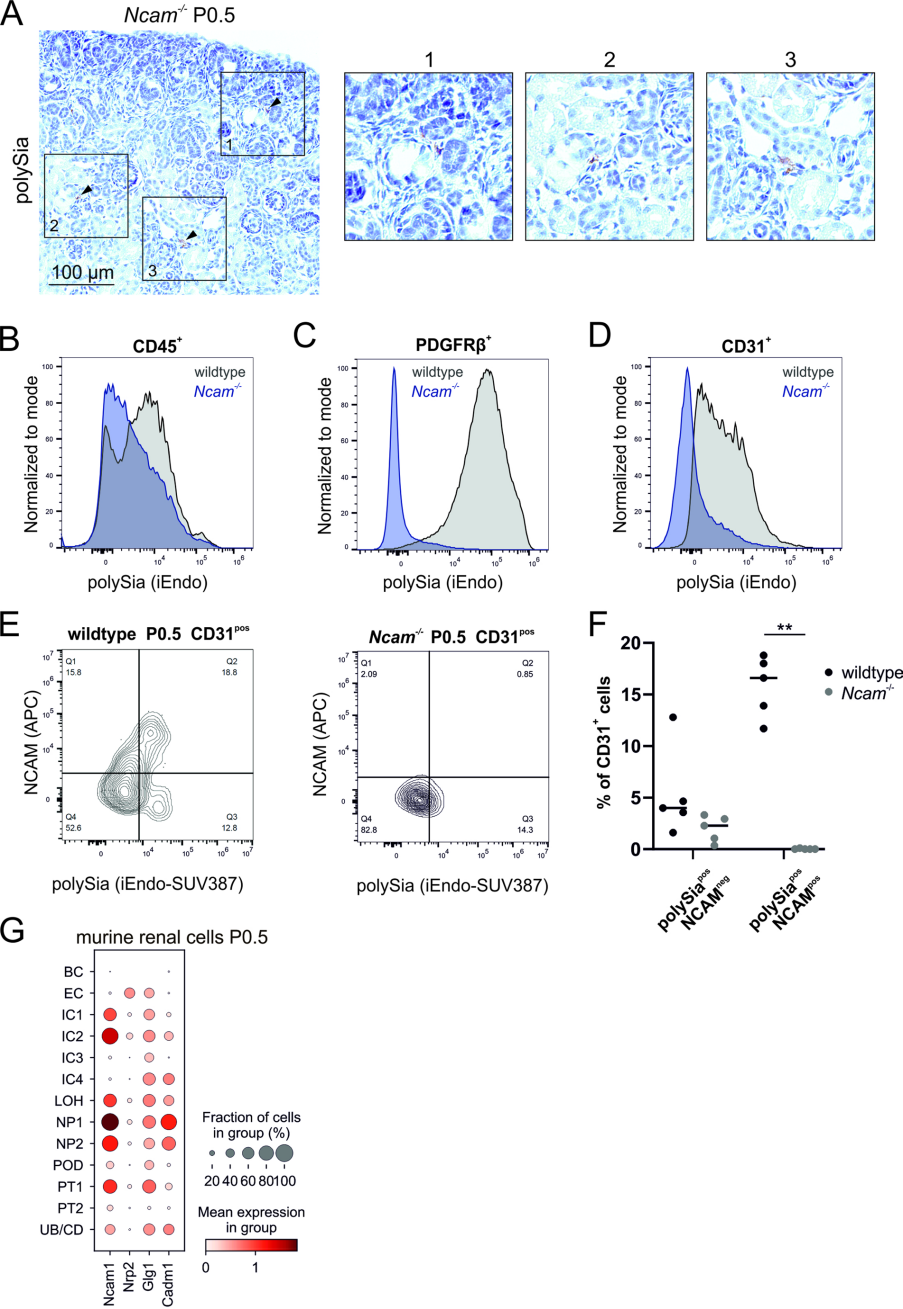
PolySia expression in *Ncam*^*−/−*^ kidney. **(A)** 735 immunohistology on renal sections of newborn *Ncam*^*−/−*^ mice. A representative segment of the cortical area is depicted with single positively stained cells (arrowheads). Three enlarged micrographs are shown to better appreciate stained cells. **(B-D)** Histograms of spectral flow cytometry analysis of single cell suspensions from P3 wildtype and *Ncam*^*−/−*^ kidneys. Cells were stained for polySia with inactive endosialidase (iEndo) and for renal cell type markers: **(B)** immune cells (CD45), **(C)** interstitial cells (PDGFRβ) and **(D)** endothelial cells (CD31). The gating strategy for the spectral flow cytometry analysis is the same as described before and depicted in Supplemental Figure S8A-E. **(E)** Contour plot of P0.5 kidney CD31-positive endothelial cells, that were analysed regarding NCAM and polySia expression from wildtype and *Ncam*^*−/−*^ mice. **(F)** Frequencies of the identified populations shown in (E) in wildtype and *Ncam*^*−/−*^ mice. Each data point represent one biological replicate. About 15% of renal endothelial cells express polySia and NCAM in wildtype mice, a frequency significantly reduced in *Ncam*^*−/−*^ mice (Nonparametric Mann–Whitney test, ** *p* < 0.01, N = 5 mice per genotype). In both genotypes, less than 5% of endothelial cells are polySia-positive but lack NCAM. **(G)** Evaluation of gene expression values of known polySia protein carriers in different renal cell types in P0 wildtype mice. Single cell RNAseq data (Accession no. GSM4648414) was obtained from Naganuma et al. (2021) [37]. LOH: loop of Henle, IC: interstitial cell, EC: endothelial cell, NP: nephron progenitor, PT: proximal tubule, UB: ureteric bud tip, CD: collecting duct, POD: podocyte, BC: blood cell. *Ncam1 or NCAM1: neural cell adhesion molecule, Nrp2 or NRP2: neuropilin-2, Glg1 or GLG1: E-selectin ligand-1/Golgi Glycoprotein 1, Cadm1 or CADM1: SynCAM 1/Cell adhesion molecule 1*

In *Ncam*^*−/−*^ kidney, glomerular tuft areas were significantly decreased (Fig. 2B). We observed a reduced number of total glomerular cells (Fig. 2D), which is attributable to a reduced number of ERG-positive glomerular endothelial cells (Fig. 2L-N), as seen in *St8sia2*^*−/−*^* St8sia4*^*−/−*^ kidneys (Fig. 2B, D, I-K). This indicates that NCAM might be the main polySia carrier in the developing kidney. Likewise, at P0.5 the polySia signal was almost completely lost in *Ncam*^*−/−*^ kidneys. Careful inspection of immunohistological staining with DAB, however, revealed a few polySia-positive cells spread across the tissue, mainly localized in the tubulointerstitial space in groups of one or two positive cells (Fig. 5A). Spectral flow cytometry analysis of wildtype and *Ncam*^*−/−*^ kidney cell suspensions at age P3 identified subsets of polySia-positive CD45-positive leukocytes in both genotypes (Fig. 5B), in line with previous reports demonstrating polySia on immune cells as a posttranslational modification of C–C motif chemokine receptor 7 (CCR7) [27] and Neuropilin 2 [52]. In contrast, polySia staining on PDGFRβ^+^ interstitial cells (Fig. 5C) was abolished in *Ncam*^*−/−*^ kidneys, indicating that NCAM is the major polySia carrier on this cell population in newborn kidney. When we analysed CD31^+^- polySia^+^ double-positive renal cells, we observed that a subfraction of the CD31^+^ endothelial cells remained polySia-positive in *Ncam*^*−/−*^ kidneys (Fig. 5D). A detailed analysis of CD31^+^ endothelial cells in the kidney of P0.5 wildtype mice confirmed the presence of two subpopulations of polySia^+^ CD31^+^ double-positive cells: an NCAM-positive and a smaller, NCAM-negative subpopulation (Fig. 5E, left panel). In line with this, most but not all of the CD31^+^ endothelial cells in *Ncam*^*−/−*^ kidney were polySia-negative (Fig. 5E, right panel). In kidneys of P0.5 wildtype mice, less than 5% of the CD31^+^ polySia^+^ endothelial cells were NCAM-negative, and a similar percentage of CD31^+^ polySia^+^ endothelial cells was observed in *Ncam*^*−/−*^ kidneys (Fig. 5F). About 15% of CD31^+^ polySia^+^ endothelial cells in wildtype mice were also NCAM-positive. These results, together with the phenotypic similarities between kidneys of *Ncam*^*−/−*^ and *St8sia2*^*−/−*^* St8sia4*^*−/−*^ mice, indicate that NCAM is the major but not the only polySia carrier in endothelial cells. Of the known polySia carriers neuropilin-2 (*Nrp2*) [52], E-selectin ligand-1/Golgi Glycoprotein 1 (*Glg1*) [53] and SynCAM 1/Cell adhesion molecule 1 (*Cadm1*) [54], only the former two are expressed in renal endothelial cells at E14.5 (Supplemental Fig. S9A) and P0.5 (Fig. 5G), which makes them promising candidates as alternative polySia carrier on endothelial cells. In order to investigate the species conservation of the pattern of polysialyltransferase and known polySia carriers’ gene expression in humans, we analysed a publicly available dataset from human fetal kidney at post-conception weeks 9–12 [38]. The comparison further revealed the conserved expression of the polysialyltransferase genes in subsets of interstitial cells, endothelial cells and mesenchyme, which gives rise to nephron progenitor cells. *Ncam* expression was conserved in fibroblasts, stroma progenitors and mesenchyme (Supplemental Fig. S9B, C). Additionally, the conservation of other known polySia protein carriers like Neuropilin-2 and ESL-1 in murine and human renal cells deserves further attention (Supplemental Fig. S9A, D).

### Polysialylated endothelial cells are more susceptible to VEGF-A signalling and receptor activation

**Fig. 6:**
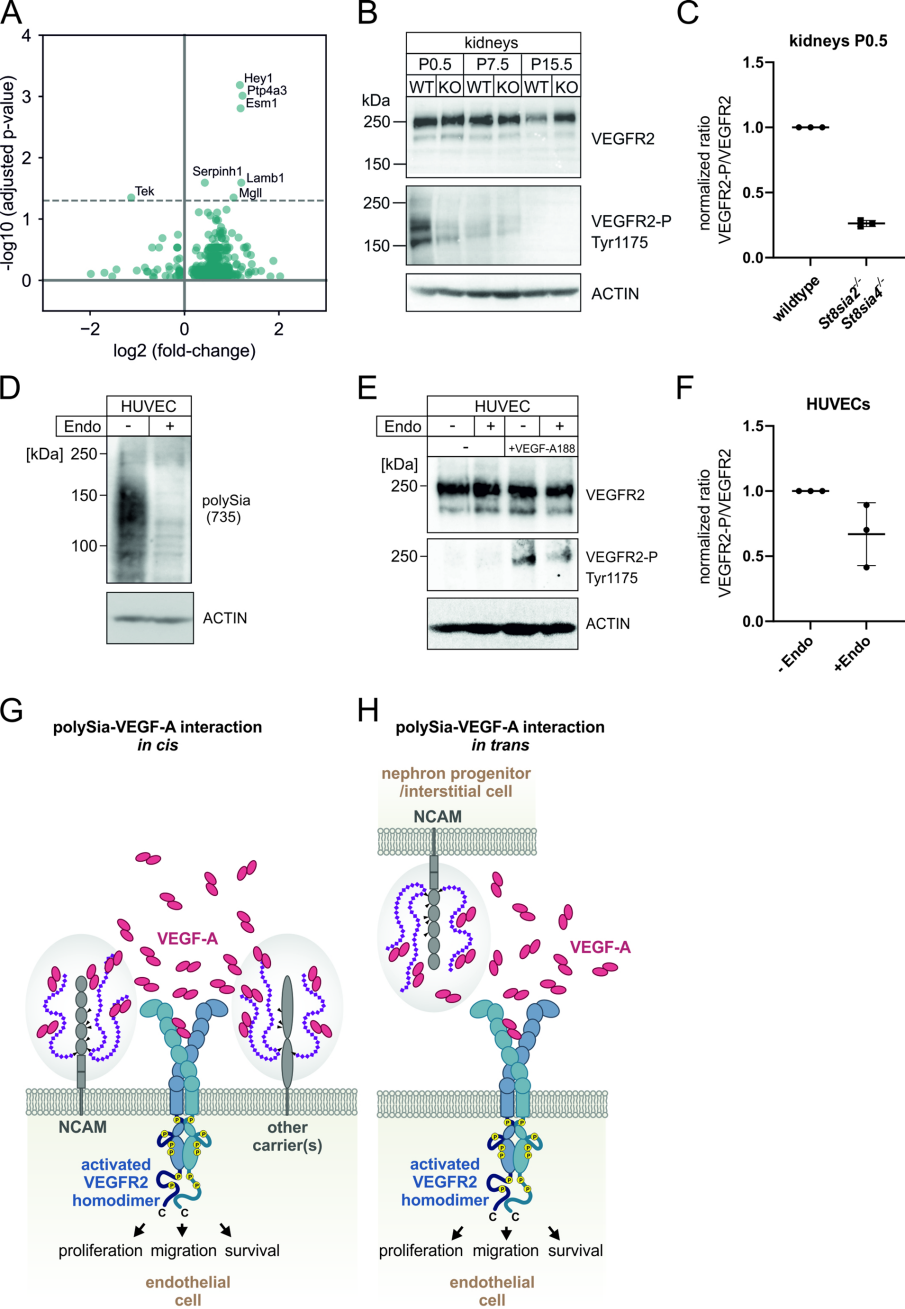
Impact of polySia on VEGF-A mediated signalling and proposed model of polySia-VEGF-A interaction. **(A)** Differential gene expression analysis between *St8sia4* + and *St8sia4*- endothelial cells extracted from the scRNAseq data set from Naganuma et al. (2021) from newborn mouse kidney. Volcano plot with -log_10_ (adj. *p*-values) plotted against the log_2_ (fold-change) is shown. Dotted line represents the significance level (adj. *p*-value < 0.05). Significantly regulated genes are labelled. *St8sia4* is not displayed in the volcano plot for reasons of clarity. **(B)** Western blot analysis of kidney homogenates from wildtype (WT) and *St8sia2*^−/−^*St8sia4*^−/−^ (KO) mice and different postnatal (P) time points. Expression of VEGFR2 and phosphorylation status of Tyr1175 of VEGFR2 is shown. Actin staining served as loading control. 25 µg total protein were loaded per lane. Representative immunoblot from n = 3 individuals is shown. **(C)** Western blot quantification of P0.5 kidney homogenates from wildtype and *St8sia2*^−/−^*St8sia4*^−/−^ mice. Samples from three wildtype and *St8sia2*^−/−^*St8sia4*^−/−^ mice were analysed pairwise on three individual Western blots. For each Western blot, signal ratios of phosphorylated VEGFR2 to total VEGFR2 protein were calculated and normalized to the wildtype. Data are shown as single values with means, and the standard deviation is shown for *St8sia2*^−/−^*St8sia4*^−/−^. **(D)** PolySia immunoblot of HUVECs treated w/o endosialidase F. Actin staining served as loading control. 10 µg total protein were loaded per lane. **(E)** Western blot analysis of HUVEC lysates w/o endosialidase F (Endo) treatment and w/o subsequent VEGF-A188 stimulation. PolySia was stained with 735 antibody. Expression of VEGFR2 and phosphorylation status of Tyr1175 of VEGFR2 are displayed. Actin staining served as loading control. 8 µg (735, VEGFR2-P) and 4 µg (VEGFR2, ACTIN) total protein were loaded per lane. A representative immunoblot from n = 3 independent experiments is shown. **(F)** Western blot quantification of HUVEC homogenates after treatment w/o endosialidase F and incubation with VEGF-A188. Samples from three independent experiments were analysed pairwise on three individual Western blots. For each Western blot, signal ratios of phosphorylated VEGFR2 to total VEGFR2 protein were calculated and normalized to the control (without endosialidase F treatment). Data are shown as single values with means, and the standard deviation is shown for endosialidase F treated cells. **(G)** PolySia (purple) on NCAM (grey) and/or other carrier protein(s) expressed on the endothelial cell surface interacts with VEGF-A (red) in close proximity to VEGFR2 (blue, with phosphorylation sites in yellow) and acts as a co-receptor to facilitate VEGF-A binding. PolySia-NCAM and VEGFR2 are in *cis* position. **(H)** PolySia-NCAM on nephron progenitor cells and/or interstitial cells are in *trans* position to VEGFR2 on endothelial cells. The polySia-VEGF-A interaction promotes the formation of a VEGF-A gradient. A combination of these two proposed models is most likely

To investigate potential differences in polySia-positive and negative renal endothelial cells, we assessed newborn kidney scRNAseq data from Naganuma et al. [37]. Differential gene expression analysis between *St8sia4*-positive and *St8sia4*-negative endothelial cells identified significantly increased expression of six genes and decreased expression of one gene (Fig. 6A and Supplemental File 2). The three most prominently upregulated genes in *St8sia4* positive ECs were *Hey1*, *Ptp4a3* and *Esm1,* all known to be associated with VEGF-A signalling. The Notch signalling target gene *Hey1* is required for vascular development and is expressed in developing nephrons [55]. The transcription of the protein tyrosine phosphatase PRL-3, a known angiogenesis regulator in endothelial cells [56], encoded by *Ptp4a3,* is induced by VEGF-A in HUVECs [57] and enables endothelial cell motility in vitro by the promotion of pro-angiogenic VEGF-A induced signalling [58]. The expression of Endothelial cell-specific molecule 1 encoded by *Esm-1,* a marker of endothelial cell activation, is also upregulated in different in vitro models of VEGF-A induced angiogenesis [59–61]. *Tek*, which encodes for the Angiopoietin-1 receptor TIE2, was significantly downregulated in *St8sia4* positive ECs, which has also been observed after VEGF-A stimulation in a cell culture model [62]. This combination of differentially regulated genes implicates the activation of VEGF-A-signalling and endothelial cell motility in polySia-positive cells. Next, we asked, whether VEGFR2 activation is altered in polySia-negative mice. In line with the results at transcriptional level (Supplemental Fig. S3), polySia-deficiency did not affect the VEGFR2 expression level (Fig. 6B). VEGFR2 activation was examined by monitoring the phosphorylation at Tyr1175, which is implicated in the activation of SHB-, PI3K- and NCK- mediated pathways that induce endothelial cell permeability, proliferation and migration [63, 64]. We observed decreased VEGFR2 phosphorylation in kidneys of polySia-negative mice by more than 70% compared to control during nephron development at time point P0.5 (Fig. 6B and C), indicating reduced receptor activation. To investigate whether polysialylation of endothelial cells directly affects VEGFR2 activation, we utilized HUVEC as a cell culture model and first confirmed the presence of polySia by Western blotting (Fig. 6D). Cell treatment with endosialidase F before immunoblotting abolished the polySia signal. Next, we added recombinantly expressed VEGF-A188 to serum starved HUVECs with and without endosialidase F pretreatment (Fig. 6E). VEGFR2 expression level did change neither after endosialidase F treatment nor after stimulation with VEGF-A188 (Fig. 6E). Receptor phosphorylation, however, was only detected after stimulation with VEGF-A, and was reduced in polySia-deficient cells (Fig. 6E and F). In summary, polysialylation increases VEGF-A mediated VEGFR2 signalling in HUVEC cells in vitro, highlighting the mechanistic cellular role of the newly described VEGF-A188 and polySia interaction.

## Discussion

In this study, we identified the linear carbohydrate homopolymer polySia as a new player in the intricate system regulating VEGF-A bioavailability. Loss of this interaction diminished VEGFR2 activation and impaired glomerular microvasculature formation during murine kidney maturation.

The crucial role of precisely balanced VEGF-A bioavailability for development and tissue homeostasis is illustrated by mouse models with altered global VEGF-A expression levels [2, 3, 65]. Experimental reduction of global VEGF-A levels in newborn mice leads to the presence of abnormal glomeruli, demonstrating that the development of the glomerular microvasculature is specifically sensitive to VEGF-A levels. Fine-tuning of balanced VEGF-A bioavailability includes alternative splicing, transcriptional and translational regulation, proteolytic cleavage as well as differential extracellular localization, due to isoform-specific interaction with cell surface glycoconjugates and co-receptors.

Here, we showed using horizontal native PAGE and an ELISA-based binding assay that murine VEGF-A188, but not VEGF-A165 and -A120, interact with the polyanionic glycan homopolymer polySia (Fig. 3C–F). In comparison to VEGF-A164, characterized by a heparan sulfate (HS) binding domain in exon 7, VEGF-A188 harbours an additional polybasic domain in exon 6. This implicates an electrostatic interaction of positively charged amino acids (see Fig. 3A) with the negatively charged polySia. Comparative modelling strategies and molecular dynamics simulations identified exon 6 encoded polybasic domain as a highly disordered flexible polycation, predestined to mediate molecular interactions [66]. In line with this, we observed polySia binding only for the exon 6-containing isoform VEGF-A188. Using a native PAGE assay similar to our approach, Strubl and co-workers previously reported an interaction of human VEGF-A165 with polySia [15]. However, in the mentioned study, only this isoform was analysed and a decrease in electrophoretic mobility towards the anode upon pre-incubation with polySia was observed, rather than the expected increase as seen in for the histone-polySia interaction used as a positive control in both studies. Therefore, more detailed studies are needed that analyse the binding of human VEGF-A to polySia, including all isoforms and using different binding assays.

The interaction of murine VEGF-A188 to polySia could be assigned to residues encoded in exon 6. This domain also constitutes a second HS binding domain and increases HS binding affinity from moderate in VEGF-A164 to high in VEGF-A188, known to create locally high VEGF-A gradients [67–69]. The only partially overlapping binding sites of HS and polySia suggest differences in the mode of molecular interaction, potentially influenced by the 3D structure and the negative charge of the glycopolymers. Based on the MST data presented in the current study, the binding affinities of VEGF-A188 for HS and polySia were in the µM range. The affinity of VEGF-A188 to the glycopolymers is thus significantly lower than the affinity of VEGF-A to its receptor VEGFR2, which is in the nM range as determined by different methods for VEGF-A165 [70–72]. HS has been shown to support VEGF-A165 signalling by promoting complex formation with VEGFR2 *in cis* [73], as well as *in trans* [74]. Since we found that the affinity of VEGF-A188 is 20-fold lower for polySia than for HS, it is possible that the two polysaccharides build instructive VEGF-A188 gradients. In light of our data (Fig. 1) showing the cell type specific spatiotemporal polySia expression pattern, it is tempting to speculate that the regulation of VEGF-A188 signalling is achieved by the spatiotemporal expression pattern of polySia. PolySia may be a potential player to fine tune VEGF-A dependent responses in endothelial cells (Fig. 6G, H). It has been shown that VEGF-A isoforms have different receptor affinities [75], trigger specific signalling cascades [76] and differentially regulate endothelial cell behaviour [76]. Endothelial cells may use a polySia-based mechanism to ensure VEGF-A isoform specific responses during development.

In the polySia-deficient mice, we observed a reduced glomerular tuft size in the mature kidney. This was a consequence of reduced endothelial cell numbers, indicating a contribution of polySia to the development of the glomerular microvasculature. This process is mediated by VEGF-A-dependent endothelial cell invasion into the vascular cleft (Fig. 3G) and subsequent vascular network formation [4]. Podocyte-specific inactivation of only one VEGF-A allele leads to severe glomerular defects, a phenotype more serious but similar to polySia-deficient mice with regard to reduced glomerular endothelial cell numbers [5]. The polySia binding VEGF-A isoform was expressed in a subset of nephron progenitor cells and infiltrating endothelial cells, and thus might contribute to both, the attraction of endothelial precursor cells in the developing nephron and the differentiation to the glomerular plexus. Importantly, since no genotype-specific differences in VEGF-A and VEGFR2 expression levels were observed, the endothelial cell phenotype is most likely attributed to a loss of the polySia-VEGF-A interaction. For efficient organ vascularization, matrix-binding properties of VEGF-A164 and -A188 are essential by generating growth factor gradients [69, 77]. Mice expressing only the freely diffusible VEGF-A120 isoform develop multiple defects including impaired renal function [69, 78]. While matrix and cell surface association of the longer VEGF-A isoforms has exclusively been attributed to the interaction with ubiquitously expressed HSPGs [79], our study highlights that polySia further fosters VEGF-A immobilisation and sensing. In contrast to the omnipresent HS, polySia shows a strictly regulated temporal and spatial expression pattern in selected cell types [80, 81], which underlines the modulatory role of this sialic acid polymer in VEGF-A availability. It is important to emphasize that this narrowly defined role of polysialylation in kidney development differs from the much broader functions of the virtually ubiquitous decoration of cell surface glycoconjugates with one or two sialic acids. The major changes of glycocalyx composition in genetic mouse models with impaired sialylation lead to impaired renal filtration barrier development and function, and these mice die postnatally due to kidney failure [20, 31]. Recently, Ferrara and colleagues [82] showed that application of the monosaccharide mannosamine (ManN) leads to a general reduction of cell surface glycosylation, activates stress pathways, and shows additivity with VEGF in promoting angiogenesis. However, in contrast to the direct impact of VEGF– polySia complex formation on VEGFR2 activation as shown in the current study, the ManN effects on endothelial cells were independent of VEGFR2 signalling [82].

ScRNAseq data from neonatal showcased biological differences related to VEGF-A signalling between polySia-positive and polySia-negative endothelial cells. A motility promoting role of polySia is already known from dendritic cells and neuronal cells during immune response as well as brain development [24, 27]. The reduced VEGFR2 phosphorylation observed in polySia-deficient HUVECs after stimulation with VEGF-A188 provided clear evidence that polySia assists in growth factor sensing and contributes to VEGFR2 activation in endothelial cells.

Significant glomerulogenesis deficits only manifested in polySia deficient mice due to simultaneous genetic inactivation of both polysialyltransferases. While the majority of polySia in murine kidney was synthesized by ST8SIA2 in subsets of nephron progenitor and interstitial cells, ST8SIA4 was the prominent enzyme in a subset of endothelial cells and immune cells. This observation implies a specific biological function of polySia in subsets of both cell types, endothelial cells and adjacent cells, like nephron progenitors and interstitial cells (see model in Fig. 6G, H). However, since loss of polySia in both cell types was required for the developmental deficits in mice, a compensatory effect cannot be excluded.

While NCAM is the major polySia carrier in the developing murine kidney, it is not the only one on endothelial cells. According to our analyses of scRNAseq data “from mice and human”, *Ncam* expression in endothelial cells is not conserved in humans [38, 83], arguing for not yet identified polySia carrier(s) in this cell type in mouse and human. Interestingly, we found several cell types in human embryonic kidneys to express a polysialyltransferase, including endothelial cells. These observations highlight the possible translational applications of our findings, regarding the tuning of isoform specific signalling through polySia, in human kidney development.

PolySia is known to capture a limited number of biologically active molecules, including FGF2 [25], brain-derived neurotrophic factor (BDNF) [26], the neurotransmitter dopamine [84], and CCL21 [27]. However, a contribution of these signalling molecules to renal deficits in polySia-deficient mice seemed unlikely. In mouse models with disturbed FGF2 [85] or dopamine signalling [86] a renal phenotype is not observed and glomerular deficits in animal models with impaired CCL21 [87, 88] and BDNF signal pathways [89] do not affect endothelial cells.

We identified a biological function for polySia during kidney development based on its role as binding partner for the pro-angiogenic isoform VEGF-A188. Loss of this interaction impairs glomerular microvasculature formation in end organ maturation. Although we confined our analysis to the role of polySia in development, our findings might contribute to determine critical steps in microvascular reconstruction. Expanding the knowledge on factors involved in glomerular capillary tuft development is of great clinical interest and may offer the chance to further improve glomerular remodelling and regeneration for therapeutical application, e.g. to regain microvascular repair after renal injury.

## Electronic Supplementary Material

Below is the link to the electronic supplementary material.

Supplementary Material 1

Supplementary Material 2

Supplementary Material 3

## Data Availability

The analytical code for scRNAseq data evaluation and the list of differentially expressed genes are provided in the Supplemental files 2 and 3.

## Abbreviations

DP: Degree of polymerization
bFGF 2: Fibroblast growth factor 2
H&E: Hematoxylin & eosin
HS: Heparan sulfate
HSPG: Heparan sulfate proteoglycans
HUVEC: Human umbilical vein endothelial cells
NZ: Nephrogenic zone
PDGFRβ^+^: Platelet derived growth factor receptor beta
polySia: Polysialic acid
scRNAseq: Single cell RNA sequencing
VEGF-A: Vascular endothelial growth factor A
VEGFR: VEGF receptor

## References

[1] M Shibuya 2011 Vascular endothelial growth factor (VEGF) and its receptor (VEGFR) signaling in angiogenesis: a crucial target for anti- and pro-angiogenic therapies Genes Cancer 2 12 1097 1105 10.1177/1947601911423031 22866201 10.1177/1947601911423031 PMC3411125

[2] N Ferrara K Carver-Moore H Chen M Dowd L Lu KS O'Shea L Powell-Braxton KJ Hillan MW Moore 1996 Heterozygous embryonic lethality induced by targeted inactivation of the VEGF gene Nature 380 6573 439 442 10.1038/380439a0 8602242 10.1038/380439a0

[3] P Carmeliet V Ferreira G Breier S Pollefeyt L Kieckens M Gertsenstein M Fahrig A Vandenhoeck K Harpal C Eberhardt C Declercq J Pawling L Moons D Collen W Risau A Nagy 1996 Abnormal blood vessel development and lethality in embryos lacking a single VEGF allele Nature 380 6573 435 439 10.1038/380435a0 8602241 10.1038/380435a0

[4] V Eremina SE Quaggin 2004 The role of VEGF-A in glomerular development and function Curr Opin Nephrol Hypertens 13 1 9 15 15090854 10.1097/00041552-200401000-00002

[5] V Eremina M Sood J Haigh A Nagy G Lajoie N Ferrara HP Gerber Y Kikkawa JH Miner SE Quaggin 2003 Glomerular-specific alterations of VEGF-A expression lead to distinct congenital and acquired renal diseases J Clin Invest 111 5 707 716 10.1172/JCI17423 12618525 10.1172/JCI17423 PMC151905

[6] B Yang DF Cross M Ollerenshaw BA Millward AG Demaine 2003 Polymorphisms of the vascular endothelial growth factor and susceptibility to diabetic microvascular complications in patients with type 1 diabetes mellitus J Diabetes Complicat 17 1 1 6 10.1016/s1056-8727(02)00181-2 10.1016/s1056-8727(02)00181-2 12505748

[7] CS Bartlett M Jeansson SE Quaggin 2016 Vascular Growth Factors and Glomerular Disease Annu Rev Physiol 78 437 461 10.1146/annurev-physiol-021115-105412 26863327 10.1146/annurev-physiol-021115-105412 PMC6450387

[8] RS Kerbel 2008 Tumor angiogenesis N Engl J Med 358 19 2039 2049 10.1056/NEJMra0706596 18463380 10.1056/NEJMra0706596 PMC4542009

[9] N Ferrara AP Adamis 2016 Ten years of anti-vascular endothelial growth factor therapy Nat Rev Drug Discov 15 6 385 403 10.1038/nrd.2015.17 26775688 10.1038/nrd.2015.17

[10] L Perez-Gutierrez N Ferrara 2023 Biology and therapeutic targeting of vascular endothelial growth factor A Nat Rev Mol Cell Biol 24 11 816 834 10.1038/s41580-023-00631-w 37491579 10.1038/s41580-023-00631-w

[11] F Shalaby J Rossant TP Yamaguchi M Gertsenstein XF Wu ML Breitman AC Schuh 1995 Failure of blood-island formation and vasculogenesis in Flk-1-deficient mice Nature 376 6535 62 66 10.1038/376062a0 7596435 10.1038/376062a0

[12] GH Fong J Rossant M Gertsenstein ML Breitman 1995 Role of the Flt-1 receptor tyrosine kinase in regulating the assembly of vascular endothelium Nature 376 6535 66 70 10.1038/376066a0 7596436 10.1038/376066a0

[13] I Zachary G Gliki 2001 Signaling transduction mechanisms mediating biological actions of the vascular endothelial growth factor family Cardiovasc Res 49 3 568 581 10.1016/s0008-6363(00)00268-6 11166270 10.1016/s0008-6363(00)00268-6

[14] KA Houck DW Leung AM Rowland J Winer N Ferrara 1992 Dual regulation of vascular endothelial growth factor bioavailability by genetic and proteolytic mechanisms J Biol Chem 267 36 26031 26037 1464614

[15] S Strubl U Schubert A Kuhnle A Rebl N Ahmadvand S Fischer KT Preissner SP Galuska 2018 Polysialic acid is released by human umbilical vein endothelial cells (HUVEC) in vitro Cell Biosci 8 64 10.1186/s13578-018-0262-y 30555678 10.1186/s13578-018-0262-y PMC6288938

[16] K Mindler E Ostertag T Stehle 2021 The polyfunctional polysialic acid: A structural view Carbohydr Res 507 108376 10.1016/j.carres.2021.108376 34273862 10.1016/j.carres.2021.108376

[17] E Ong J Nakayama K Angata L Reyes T Katsuyama Y Arai M Fukuda 1998 Developmental regulation of polysialic acid synthesis in mouse directed by two polysialyltransferases PST and STX Glycobiology 8 4 415 424 10.1093/glycob/8.4.415 9499389 10.1093/glycob/8.4.415

[18] H Hildebrandt A Dityatev 2015 Polysialic acid in brain development and synaptic plasticity Top Curr Chem 366 55 96 10.1007/128_2013_446 23715636 10.1007/128_2013_446

[19] PM Lackie C Zuber J Roth 1994 Polysialic acid of the neural cell adhesion molecule (N-CAM) is widely expressed during organogenesis in mesodermal and endodermal derivatives Differentiation 57 2 119 131 8070624 10.1046/j.1432-0436.1994.5720119.x

[20] B Weinhold M Sellmeier W Schaper L Blume B Philippens E Kats U Bernard SP Galuska H Geyer R Geyer K Worthmann M Schiffer S Groos R Gerardy-Schahn AK Münster-Kühnel 2012 Deficits in sialylation impair podocyte maturation J Am Soc Nephrol 23 8 1319 1328 10.1681/ASN.2011090947 22745475 10.1681/ASN.2011090947 PMC3402283

[21] J Roth DJ Taatjes D Bitter-Suermann J Finne 1987 Polysialic acid units are spatially and temporally expressed in developing postnatal rat kidney Proc Natl Acad Sci USA 84 7 1969 1973 3470771 10.1073/pnas.84.7.1969 PMC304563

[22] PM Lackie C Zuber J Roth 1991 Expression of polysialylated N-CAM during rat heart development Differentiation 47 2 85 98 1955110 10.1111/j.1432-0436.1991.tb00226.x

[23] A Mayerhofer G Lahr K Seidl B Eusterschulte A Christoph M Gratzl 1996 The neural cell adhesion molecule (NCAM) provides clues to the development of testicular Leydig cells J Androl 17 3 223 230 8792212

[24] B Weinhold R Seidenfaden I Rockle M Muhlenhoff F Schertzinger S Conzelmann JD Marth R Gerardy-Schahn H Hildebrandt 2005 Genetic ablation of polysialic acid causes severe neurodevelopmental defects rescued by deletion of the neural cell adhesion molecule J Biol Chem 280 52 42971 42977 10.1074/jbc.M511097200 16267048 10.1074/jbc.M511097200

[25] S Ono M Hane K Kitajima C Sato 2012 Novel regulation of fibroblast growth factor 2 (FGF2)-mediated cell growth by polysialic acid J Biol Chem 287 6 3710 3722 10.1074/jbc.M111.276618 22158871 10.1074/jbc.M111.276618 PMC3281739

[26] Y Kanato K Kitajima C Sato 2008 Direct binding of polysialic acid to a brain-derived neurotrophic factor depends on the degree of polymerization Glycobiology 18 12 1044 1053 10.1093/glycob/cwn084 18796648 10.1093/glycob/cwn084

[27] E Kiermaier C Moussion CT Veldkamp R Gerardy-Schahn I Vries de LG Williams GR Chaffee AJ Phillips F Freiberger R Imre D Taleski RJ Payne A Braun R Forster K Mechtler M Mühlenhoff BF Volkman M Sixt 2016 Polysialylation controls dendritic cell trafficking by regulating chemokine recognition Science 351 6269 186 190 10.1126/science.aad0512 26657283 10.1126/science.aad0512 PMC5583642

[28] K Angata JM Long O Bukalo W Lee A Dityatev A Wynshaw-Boris M Schachner M Fukuda JD Marth 2004 Sialyltransferase ST8Sia-II assembles a subset of polysialic acid that directs hippocampal axonal targeting and promotes fear behavior J Biol Chem 279 31 32603 32613 10.1074/jbc.M403429200 15140899 10.1074/jbc.M403429200

[29] M Eckhardt O Bukalo G Chazal L Wang C Goridis M Schachner R Gerardy-Schahn H Cremer A Dityatev 2000 Mice deficient in the polysialyltransferase ST8SiaIV/PST-1 allow discrimination of the roles of neural cell adhesion molecule protein and polysialic acid in neural development and synaptic plasticity J Neurosci 20 14 5234 5244 10.1523/JNEUROSCI.20-14-05234.2000 10884307 10.1523/JNEUROSCI.20-14-05234.2000 PMC6772332

[30] H Cremer R Lange A Christoph M Plomann G Vopper J Roes R Brown S Baldwin P Kraemer S Scheff 1994 Inactivation of the N-CAM gene in mice results in size reduction of the olfactory bulb and deficits in spatial learning Nature 367 6462 455 459 10.1038/367455a0 8107803 10.1038/367455a0

[31] KM Niculovic L Blume H Wedekind E Kats I Albers S Groos M Abeln J Schmitz E Beuke JH Bräsen A Melk M Schiffer B Weinhold AK Münster-Kühnel 2019 Podocyte-specific sialylation-deficient mice serve as model for human focal segmental glomerulosclerosis J Am Soc Nephrol 30 1021 1035 31040189 10.1681/ASN.2018090951 PMC6551790

[32] C Weisgerber M Husmann M Frosch C Rheinheimer W Peuckert I Gorgen D Bitter-Suermann 1990 Embryonic neural cell adhesion molecule in cerebrospinal fluid of younger children: age-dependent decrease during the first year J Neurochem 55 6 2063 2071 10.1111/j.1471-4159.1990.tb05796.x 2230809 10.1111/j.1471-4159.1990.tb05796.x

[33] LJ Schroder H Thiesler L Gretenkort TM Mollenkamp M Stangel V Gudi H Hildebrandt 2023 Polysialic acid promotes remyelination in cerebellar slice cultures by Siglec-E-dependent modulation of microglia polarization Front Cell Neurosci 17 1207540 10.3389/fncel.2023.1207540 37492129 10.3389/fncel.2023.1207540 PMC10365911

[34] T McLellan 1982 Electrophoresis buffers for polyacrylamide gels at various pH Anal Biochem 126 1 94 99 10.1016/0003-2697(82)90113-0 7181120 10.1016/0003-2697(82)90113-0

[35] CJ Wienken P Baaske U Rothbauer D Braun S Duhr 2010 Protein-binding assays in biological liquids using microscale thermophoresis Nat Commun 1 100 10.1038/ncomms1093 20981028 10.1038/ncomms1093

[36] D Schwarzer K Stummeyer T Haselhorst F Freiberger B Rode M Grove T Scheper M Itzstein von M Muhlenhoff R Gerardy-Schahn 2009 Proteolytic release of the intramolecular chaperone domain confers processivity to endosialidase F J Biol Chem 284 14 9465 9474 10.1074/jbc.M808475200 19189967 10.1074/jbc.M808475200 PMC2666599

[37] H Naganuma K Miike T Ohmori S Tanigawa T Ichikawa M Yamane M Eto H Niwa A Kobayashi R Nishinakamura 2021 Molecular detection of maturation stages in the developing kidney Dev Biol 470 62 73 10.1016/j.ydbio.2020.11.002 33197428 10.1016/j.ydbio.2020.11.002

[38] BJ Stewart JR Ferdinand MD Young TJ Mitchell KW Loudon AM Riding N Richoz GL Frazer JUL Staniforth FA Vieira Braga RA Botting DM Popescu R Vento-Tormo E Stephenson A Cagan SJ Farndon K Polanski M Efremova K Green V-H Castillo Del C Guzzo G Collord L Mamanova T Aho JN Armitage ACP Riddick I Mushtaq S Farrell D Rampling J Nicholson A Filby J Burge S Lisgo S Lindsay M Bajenoff AY Warren GD Stewart N Sebire N Coleman M Haniffa SA Teichmann S Behjati MR Clatworthy 2019 Spatiotemporal immune zonation of the human kidney Science 365 6460 1461 1466 10.1126/science.aat5031 31604275 10.1126/science.aat5031 PMC7343525

[39] HJ Jennings R Roy F Michon 1985 Determinant specificities of the groups B and C polysaccharides of Neisseria meningitidis J Immunol 134 4 2651 2657 2579148

[40] J Hayrinen D Bitter-Suermann J Finne 1989 Interaction of meningococcal group B monoclonal antibody and its Fab fragment with alpha 2–8-linked sialic acid polymers: requirement of a long oligosaccharide segment for binding Mol Immunol 26 6 523 529 10.1016/0161-5890(89)90003-5 2505065 10.1016/0161-5890(89)90003-5

[41] D Schwarzer K Stummeyer R Gerardy-Schahn M Muhlenhoff 2007 Characterization of a novel intramolecular chaperone domain conserved in endosialidases and other bacteriophage tail spike and fiber proteins J Biol Chem 282 5 2821 2831 10.1074/jbc.M609543200 17158460 10.1074/jbc.M609543200

[42] RL Schnaar R Gerardy-Schahn H Hildebrandt 2014 Sialic acids in the brain: gangliosides and polysialic acid in nervous system development, stability, disease, and regeneration Physiol Rev 94 2 461 518 10.1152/physrev.00033.2013 24692354 10.1152/physrev.00033.2013 PMC4044301

[43] Y Kitamoto H Tokunaga K Tomita 1997 Vascular endothelial growth factor is an essential molecule for mouse kidney development: glomerulogenesis and nephrogenesis J Clin Invest 99 10 2351 2357 10.1172/JCI119416 9153276 10.1172/JCI119416 PMC508073

[44] SP Galuska I Oltmann-Norden H Geyer B Weinhold K Kuchelmeister H Hildebrandt R Gerardy-Schahn R Geyer M Mühlenhoff 2006 Polysialic acid profiles of mice expressing variant allelic combinations of the polysialyltransferases ST8SiaII and ST8SiaIV J Biol Chem 281 42 31605 31615 10.1074/jbc.M606516200 16940046 10.1074/jbc.M606516200

[45] A Mori Y Yang Y Takahashi M Hane K Kitajima C Sato 2020 Combinational analyses with multiple methods reveal the existence of several forms of polysialylated neural cell adhesion molecule in mouse developing brains Int J Mol Sci 21 16 892 10.3390/ijms21165892 32824359 10.3390/ijms21165892 PMC7460633

[46] B Mishra M Ohe von der C Schulze S Bian T Makhina G Loers R Kleene M Schachner 2010 Functional role of the interaction between polysialic acid and extracellular histone H1 J Neurosci 30 37 12400 12413 10.1523/JNEUROSCI.6407-09.2010 20844135 10.1523/JNEUROSCI.6407-09.2010 PMC6633434

[47] C Schell N Wanner TB Huber 2014 Glomerular development–shaping the multi-cellular filtration unit Semin Cell Dev Biol 36 39 49 10.1016/j.semcdb.2014.07.016 25153928 10.1016/j.semcdb.2014.07.016

[48] A Kobayashi MT Valerius JW Mugford TJ Carroll M Self G Oliver AP McMahon 2008 Six2 defines and regulates a multipotent self-renewing nephron progenitor population throughout mammalian kidney development Cell Stem Cell 3 2 169 181 10.1016/j.stem.2008.05.020 18682239 10.1016/j.stem.2008.05.020 PMC2561900

[49] R Menon EA Otto A Kokoruda J Zhou Z Zhang E Yoon YC Chen O Troyanskaya JR Spence M Kretzler C Cebrian 2018 Single-cell analysis of progenitor cell dynamics and lineage specification in the human fetal kidney Development 145 16 164038 10.1242/dev.164038 10.1242/dev.164038 PMC6124540 30166318

[50] A Schumacher MB Rookmaaker JA Joles R Kramann TQ Nguyen M Griensven van VLS LaPointe 2021 Defining the variety of cell types in developing and adult human kidneys by single-cell RNA sequencing NPJ Regen Med 6 1 45 10.1038/s41536-021-00156-w 34381054 10.1038/s41536-021-00156-w PMC8357940

[51] AR England CP Chaney A Das M Patel A Malewska D Armendariz GC Hon DW Strand KA Drake TJ Carroll 2020 Identification and characterization of cellular heterogeneity within the developing renal interstitium Development 147 15 190108 10.1242/dev.190108 10.1242/dev.190108 PMC7438011 32586976

[52] S Curreli Z Arany R Gerardy-Schahn D Mann NM Stamatos 2007 Polysialylated neuropilin-2 is expressed on the surface of human dendritic cells and modulates dendritic cell-T lymphocyte interactions J Biol Chem 282 42 30346 30356 10.1074/jbc.M702965200 17699524 10.1074/jbc.M702965200

[53] S Werneburg FF Buettner L Erben M Mathews H Neumann M Mühlenhoff H Hildebrandt 2016 Polysialylation and lipopolysaccharide-induced shedding of E-selectin ligand-1 and neuropilin-2 by microglia and THP-1 macrophages Glia 64 8 1314 1330 10.1002/glia.23004 27159043 10.1002/glia.23004

[54] SP Galuska M Rollenhagen M Kaup K Eggers I Oltmann-Norden M Schiff M Hartmann B Weinhold H Hildebrandt R Geyer M Mühlenhoff H Geyer 2010 Synaptic cell adhesion molecule SynCAM 1 is a target for polysialylation in postnatal mouse brain Proc Natl Acad Sci U S A 107 22 10250 10255 10.1073/pnas.0912103107 20479255 10.1073/pnas.0912103107 PMC2890427

[55] L Chen Q Al-Awqati 2005 Segmental expression of Notch and Hairy genes in nephrogenesis Am J Physiol Renal Physiol 288 5 F939 952 10.1152/ajprenal.00369.2004 15821257 10.1152/ajprenal.00369.2004

[56] M Poulet J Sirois K Boye N Uetani S Hardy T Daubon A Dubrac ML Tremblay A Bikfalvi 2020 PRL-2 phosphatase is required for vascular morphogenesis and angiogenic signaling Commun Biol 3 1 603 10.1038/s42003-020-01343-z 33097786 10.1038/s42003-020-01343-z PMC7584612

[57] J Xu S Cao L Wang R Xu G Chen Q Xu 2011 VEGF promotes the transcription of the human PRL-3 gene in HUVEC through transcription factor MEF2C PLoS ONE 6 11 e27165 10.1371/journal.pone.0027165 22073279 10.1371/journal.pone.0027165 PMC3206935

[58] MW Zimmerman KE McQueeney JS Isenberg BR Pitt KA Wasserloos GE Homanics JS Lazo 2014 Protein-tyrosine phosphatase 4A3 (PTP4A3) promotes vascular endothelial growth factor signaling and enables endothelial cell motility J Biol Chem 289 9 5904 5913 10.1074/jbc.M113.480038 24403062 10.1074/jbc.M113.480038 PMC3937659

[59] M Delehedde L Devenyns CA Maurage RR Vives 2013 Endocan in cancers: a lesson from a circulating dermatan sulfate proteoglycan Int J Cell Biol 2013 705027 10.1155/2013/705027 23606845 10.1155/2013/705027 PMC3625564

[60] E Rennel S Mellberg A Dimberg L Petersson J Botling A Ameur JO Westholm J Komorowski P Lassalle MJ Cross P Gerwins 2007 Endocan is a VEGF-A and PI3K regulated gene with increased expression in human renal cancer Exp Cell Res 313 7 1285 1294 10.1016/j.yexcr.2007.01.021 17362927 10.1016/j.yexcr.2007.01.021

[61] ME Gerritsen JE Tomlinson C Zlot M Ziman S Hwang 2003 Using gene expression profiling to identify the molecular basis of the synergistic actions of hepatocyte growth factor and vascular endothelial growth factor in human endothelial cells Br J Pharmacol 140 4 595 610 10.1038/sj.bjp.0705494 14504135 10.1038/sj.bjp.0705494 PMC1574080

[62] M Felcht R Luck A Schering P Seidel K Srivastava J Hu A Bartol Y Kienast C Vettel EK Loos S Kutschera S Bartels S Appak E Besemfelder D Terhardt E Chavakis T Wieland C Klein M Thomas A Uemura S Goerdt HG Augustin 2012 Angiopoietin-2 differentially regulates angiogenesis through TIE2 and integrin signaling J Clin Invest 122 6 1991 2005 10.1172/JCI58832 22585576 10.1172/JCI58832 PMC3366398

[63] X Wang AM Bove G Simone B Ma 2020 Molecular Bases of VEGFR-2-Mediated Physiological Function and Pathological Role Front Cell Dev Biol 8 599281 10.3389/fcell.2020.599281 33304904 10.3389/fcell.2020.599281 PMC7701214

[64] T Takahashi S Yamaguchi K Chida M Shibuya 2001 A single autophosphorylation site on KDR/Flk-1 is essential for VEGF-A-dependent activation of PLC-gamma and DNA synthesis in vascular endothelial cells EMBO J 20 11 2768 2778 10.1093/emboj/20.11.2768 11387210 10.1093/emboj/20.11.2768 PMC125481

[65] L Miquerol BL Langille A Nagy 2000 Embryonic development is disrupted by modest increases in vascular endothelial growth factor gene expression Development 127 18 3941 3946 10.1242/dev.127.18.3941 10952892 10.1242/dev.127.18.3941

[66] F Bergantino S Guariniello R Raucci G Colonna A Luca De N Normanno 1854 Costantini S (2015) Structure-fluctuation-function relationships of seven pro-angiogenic isoforms of VEGFA, important mediators of tumorigenesis Biochim Biophys Acta 5 410 425 10.1016/j.bbapap.2015.01.005 10.1016/j.bbapap.2015.01.005 25617660

[67] JE Park GA Keller N Ferrara 1993 The vascular endothelial growth factor (VEGF) isoforms: differential deposition into the subepithelial extracellular matrix and bioactivity of extracellular matrix-bound VEGF Mol Biol Cell 4 12 1317 1326 10.1091/mbc.4.12.1317 8167412 10.1091/mbc.4.12.1317 PMC275767

[68] D Krilleke A DeErkenez W Schubert I Giri GS Robinson YS Ng DT Shima 2007 Molecular mapping and functional characterization of the VEGF164 heparin-binding domain J Biol Chem 282 38 28045 28056 10.1074/jbc.M700319200 17626017 10.1074/jbc.M700319200

[69] C Ruhrberg H Gerhardt M Golding R Watson S Ioannidou H Fujisawa C Betsholtz DT Shima 2002 Spatially restricted patterning cues provided by heparin-binding VEGF-A control blood vessel branching morphogenesis Genes Dev 16 20 2684 2698 10.1101/gad.242002 12381667 10.1101/gad.242002 PMC187458

[70] BI Terman M Dougher-Vermazen ME Carrion D Dimitrov DC Armellino D Gospodarowicz P Bohlen 1992 Identification of the KDR tyrosine kinase as a receptor for vascular endothelial cell growth factor Biochem Biophys Res Commun 187 3 1579 1586 10.1016/0006-291x(92)90483-2 1417831 10.1016/0006-291x(92)90483-2

[71] J Waltenberger L Claesson-Welsh A Siegbahn M Shibuya CH Heldin 1994 Different signal transduction properties of KDR and Flt1, two receptors for vascular endothelial growth factor J Biol Chem 269 43 26988 26995 7929439

[72] CJ Peach LE Kilpatrick R Friedman-Ohana K Zimmerman MB Robers KV Wood J Woolard SJ Hill 2018 Real-time ligand binding of fluorescent VEGF-a isoforms that discriminate between VEGFR2 and NRP1 in living cells Cell Chem Biol 25 10 1208 1218 10.1016/j.chembiol.2018.06.012 30057299 10.1016/j.chembiol.2018.06.012 PMC6200776

[73] L Jakobsson J Kreuger K Holmborn L Lundin I Eriksson L Kjellen L Claesson-Welsh 2006 Heparan sulfate in trans potentiates VEGFR-mediated angiogenesis Dev Cell 10 5 625 634 10.1016/j.devcel.2006.03.009 16678777 10.1016/j.devcel.2006.03.009

[74] Y Shintani S Takashima Y Asano H Kato Y Liao S Yamazaki O Tsukamoto O Seguchi H Yamamoto T Fukushima K Sugahara M Kitakaze M Hori 2006 Glycosaminoglycan modification of neuropilin-1 modulates VEGFR2 signaling EMBO J 25 13 3045 3055 10.1038/sj.emboj.7601188 16763549 10.1038/sj.emboj.7601188 PMC1500974

[75] SB Mamer A Wittenkeller PI Imoukhuede 2020 VEGF-A splice variants bind VEGFRs with differential affinities Sci Rep 10 1 14413 10.1038/s41598-020-71484-y 32879419 10.1038/s41598-020-71484-y PMC7468149

[76] GW Fearnley GA Smith I Abdul-Zani N Yuldasheva NA Mughal S Homer-Vanniasinkam MT Kearney IC Zachary DC Tomlinson MA Harrison SB Wheatcroft S Ponnambalam 2016 VEGF-A isoforms program differential VEGFR2 signal transduction, trafficking and proteolysis Biol Open 5 5 571 583 10.1242/bio.017434 27044325 10.1242/bio.017434 PMC4874356

[77] H Gerhardt M Golding M Fruttiger C Ruhrberg A Lundkvist A Abramsson M Jeltsch C Mitchell K Alitalo D Shima C Betsholtz 2003 VEGF guides angiogenic sprouting utilizing endothelial tip cell filopodia J Cell Biol 161 6 1163 1177 10.1083/jcb.200302047 12810700 10.1083/jcb.200302047 PMC2172999

[78] V Mattot L Moons F Lupu D Chernavvsky RA Gomez D Collen P Carmeliet 2002 Loss of the VEGF(164) and VEGF(188) isoforms impairs postnatal glomerular angiogenesis and renal arteriogenesis in mice J Am Soc Nephrol 13 6 1548 1560 10.1097/01.asn.0000013925.19218.7b 12039984 10.1097/01.asn.0000013925.19218.7b

[79] A Ori MC Wilkinson DG Fernig 2011 A systems biology approach for the investigation of the heparin/heparan sulfate interactome J Biol Chem 286 22 19892 19904 10.1074/jbc.M111.228114 21454685 10.1074/jbc.M111.228114 PMC3103365

[80] TM Villanueva-Cabello LD Gutierrez-Valenzuela R Salinas-Marin DV Lopez-Guerrero I Martinez-Duncker 2021 Polysialic acid in the immune system Front Immunol 12 823637 10.3389/fimmu.2021.823637 35222358 10.3389/fimmu.2021.823637 PMC8873093

[81] H Thiesler M Kucukerden L Gretenkort I Rockle H Hildebrandt 2022 News and views on polysialic acid: from tumor progression and brain development to psychiatric disorders, neurodegeneration, myelin repair and immunomodulation Front Cell Dev Biol 10 871757 10.3389/fcell.2022.871757 35617589 10.3389/fcell.2022.871757 PMC9013797

[82] C Zhong P Li S Argade L Liu A Chilla W Liang H Xin B Eliceiri B Choudhury N Ferrara 2020 Inhibition of protein glycosylation is a novel pro-angiogenic strategy that acts via activation of stress pathways Nat Commun 11 1 6330 10.1038/s41467-020-20108-0 33303737 10.1038/s41467-020-20108-0 PMC7730427

[83] NO Lindstrom R Sealfon X Chen RK Parvez A Ransick Brandine G Sena De J Guo B Hill T Tran AD Kim J Zhou A Tadych A Watters A Wong E Lovero BH Grubbs ME Thornton JA McMahon AD Smith SW Ruffins C Armit OG Troyanskaya AP McMahon 2021 Spatial transcriptional mapping of the human nephrogenic program Dev Cell 56 16 2381 2398 10.1016/j.devcel.2021.07.017 34428401 10.1016/j.devcel.2021.07.017 PMC8396064

[84] R Isomura K Kitajima C Sato 2011 Structural and functional impairments of polysialic acid by a mutated polysialyltransferase found in schizophrenia J Biol Chem 286 24 21535 21545 10.1074/jbc.M111.221143 21464126 10.1074/jbc.M111.221143 PMC3122212

[85] J Qiao KT Bush DL Steer RO Stuart H Sakurai W Wachsman SK Nigam 2001 Multiple fibroblast growth factors support growth of the ureteric bud but have different effects on branching morphogenesis Mech Dev 109 2 123 135 10.1016/s0925-4773(01)00592-5 11731227 10.1016/s0925-4773(01)00592-5

[86] X Wang VA Villar I Armando GM Eisner RA Felder PA Jose 2008 Dopamine, kidney, and hypertension: studies in dopamine receptor knockout mice Pediatr Nephrol 23 12 2131 2146 10.1007/s00467-008-0901-3 18615257 10.1007/s00467-008-0901-3 PMC3724362

[87] B Banas M Wornle T Berger PJ Nelson CD Cohen M Kretzler J Pfirstinger M Mack M Lipp HJ Grone D Schlondorff 2002 Roles of SLC/CCL21 and CCR7 in human kidney for mesangial proliferation, migration, apoptosis, and tissue homeostasis J Immunol 168 9 4301 4307 10.4049/jimmunol.168.9.4301 11970971 10.4049/jimmunol.168.9.4301

[88] S Wurm A Steege EM Rom-Jurek CR Roeyen van A Kurtz B Banas MC Banas 2018 CCR7 Is Important for Mesangial Cell Physiology and Repair J Histochem Cytochem 66 1 7 22 10.1369/0022155417737975 29077526 10.1369/0022155417737975 PMC5761946

[89] N Endlich T Lange J Kuhn P Klemm AM Kotb F Siegerist F Kindt MT Lindenmeyer CD Cohen AW Kuss N Nath R Rettig U Lendeckel U Zimmermann K Amann S Stracke K Endlich 2018 BDNF: mRNA expression in urine cells of patients with chronic kidney disease and its role in kidney function J Cell Mol Med 22 11 5265 5277 10.1111/jcmm.13762 30133147 10.1111/jcmm.13762 PMC6201371

