# Supplementary material for: Polysialic acid regulates glomerular microvasculature formation by interaction with VEGF-A188 in mice
Source: Angiogenesis. 2025 May 24;28(3):31. doi: 10.1007/s10456-025-09984-6 (PMC12103385; doi:10.1007/s10456-025-09984-6)
Supplement: Supplementary file 1 — Supplementary Material 1 [file 10456_2025_9984_MOESM1_ESM.pdf]

Supplemental File 1

Polysialic acid regulates glomerular microvasculature formation by interaction with VEGF-A188 in mice

Authors

Kristina M. Niculovic[a,1]* (Dr.), Manuel M. Vicente[a]* (PhD), Vanessa Wittek[a]*, Elina Kats[a], Iris Albers[a], Kerstin Flächsig-Schulz[a], Ulrike Peters-Bernard[a], Anna-Carina Weiss[a] (Dr.), Hauke Thiesler[a] (Dr.), Laura S. Dräger[a], Manuel H. Taft[b] (Dr.), Anne Jörns[a] (Prof.), Hans Bakker[a] (Dr.), Herbert Hildebrandt[a] (Prof.), Martina Mühlenhoff[a] (Dr.), Birgit Weinhold[a] (Dr.), Markus Abeln[a] (Dr.), Anja K. Münster-Kühnel[a] (Dr.) #

*Equally contributing

Author affiliations

[a]Institute of Clinical Biochemistry, Hannover Medical School, 30625 Hannover, Germany

[b]Institute for Biophysical Chemistry, Hannover Medical School, 30625 Hannover, Germany

Corresponding author

#Anja K. Münster-Kühnel, Institute of Clinical Biochemistry, Hannover Medical School, Carl-Neuberg-Str. 1, 30625 Hannover, Germany, Phone: +49-511-532-9807, email: Muenster.Anja@mh-hannover.de

Supplemental File 1

Supplemental Methods

Immunohistochemistry and indirect immunofluorescence

Section RNA *in situ* hybridization

Western blot analysis of kidney and cell lysates

Quantitative real-time PCR

Cultivation of human umbilical vein endothelial cells (HUVECs)

Image acquisition

Single-cell RNA sequencing data processing

Enzyme-linked immunosorbent assay

Supplemental Figures

Supplemental Figure S1	Specificity control of 735 immunohistochemistry on renal sections.
Supplemental Figure S2	Kidney architecture markers in renal sections of wildtype and polySia-negative mice.
Supplemental Figure S3	Relative mRNA expression of *Vegfa* isoforms and *Kdr* (VEGFR2) in murine kidney.
Supplemental Figure S4	Binding assay of VEGF-A isoforms with polySia and HS as well as mono-, di-, oligo- Sia in horizontal native PAGE.
Supplemental Figure S5	Scheme of the indirect PolySia ELISA setting.
Supplemental Figure S6	*Vegf-a188* mRNA structure and illustration of the BaseScope assay principle.
Supplemental Figure S7	Polysialyltransferase and polySia expression in the nascent nephron.
Supplemental Figure S8	Spectral flow analysis of kidney single cell suspensions from newborn wildtype and knockout mice.
Supplemental Figure S9	Single Cell RNAseq analysis of mouse and human renal samples during development.

Supplemental Tables

Supplemental Table S1.	Genotyping primer
Supplemental Table S2.	Antibodies
Supplemental Table S3.	Quantitative real-time PCR primer

Supplemental Methods

Immunohistochemistry and indirect immunofluorescence

For immunohistochemistry and indirect immunofluorescence staining on paraffin-embedded kidney sections (3 µm), Target Retrieval Solution Citrate pH 6 (Dako) or 400 mM Tris HCl pH 9.0, 1 mM EDTA, 0.05% Tween was used for antigen retrieval at 100°C for 10 min or 90°C for 45 min, respectively. Blocking and primary antibody incubations were performed with either 5% skimmed milk/PBS (WT1 antibody) or 1% BSA/PBS. For visualization with DAB, endogenous peroxidase was blocked with Peroxidase-Blocking Solution (Dako) or 5% H_2O_2 in PBS prior to staining and appropriate horse reddish peroxidase coupled secondary antibodies were used. After nuclear counterstaining with hematoxylin the tissue section was mounted with Vitroclud (Vector Laboratories). For indirect immunofluorescence the primary antibody was incubated over night at 4°C or for 1 h at room temperature. Secondary antibody incubations were performed in 1% BSA/PBS for 1 h at room temperature. Endosialidase treatment was carried out with 10 µg/ml recombinantly expressed and in house purified endosialidase F (Schwarzer D. et al., (2007) J Biol Chem 282, 2821-2831) in 1% BSA/PBS for 2 h at 37°C prior to the staining procedure. Antibody dilutions and purchase numbers are listed in Supplemental Table S2.

Section RNA *in situ* hybridization

10 µm paraffin-embedded kidney sections from newborn mice were dewaxed, re-hydrated and treated with proteinase K in PBS for 5–15 min at 37°C, followed by a 5 min rinse in 0.2% glycine/PBS and two rinses of 5 min in PBS. Sections were then re-fixed for 20 min in 4% PFA/0.2% glutaraldehyde dissolved in PBS to ensure firm attachment of the

sections to the microscope slides and washed 2x in PBS for 5 min. Sections were pre-hybridized in hybridization mix without probe for 30 min - 1 h at 70°C and then hybridized with probe overnight at 70°C. Approximately 10 µl hybridization mix was applied to the sections. After hybridization, sections were rinsed in 2x SSC, washed 2x for 30 min at 65°C in 50% formamide/2x SSC, followed by three 5 min washes in PBS/Tween. Sections were transferred to an alkaline buffer (pH 9.5). Probe bound to the section was immunologically detected using anti-digoxigenin Fab fragment coupled to alkaline phosphatase and BMPurple (Roche) as chromogenic substrate. Color development was monitored and stopped with PBS, re-fixed in 4% PFA/PBS and mounted with an aqueous mounting medium.

Western blot analysis of kidney and cell lysates

Murine kidney homogenates and HUVEC cell lysates were prepared in RIPA buffer (according to Abcam) supplemented with the protease inhibitors aprotinine and leupeptin (Sigma) and a phosphatase inhibitor cocktail set V (Merck). Protein concentrations were determined with a BCA Assay (Thermo Scientific). For endosialidase treatment an aliquot of the sample was incubated with 20 µg endosialidase F per mg total protein at 37°C for 30 min prior to analysis. Lysates were supplemented with Laemmli loading buffer and equal protein amounts were separated in an 8-12% SDS-PAGE and blotted onto nitrocellulose or PVDF membrane (Merck). Membranes were incubated with primary antibodies overnight at 4°C in 3% BSA/PBS/0.1% Tween (ACTIN), 3% BSA/TBS/0.1% Tween (VEGFR2 and VEGFR2-P) or 3% skimmed milk/PBS/0.1% Tween (735 and VEGF-A) and appropriate peroxidase-coupled secondary antibodies for 1 h at room temperature. Antibodies are listed in Supplemental Table S2. Antibody staining was visualized with ECL Western blotting substrate (Thermo Fischer) or Super signal West

Femto Kit (Thermo Fischer) and documented with an Amersham imager AI680 (GE healthcare). Western blot quantification in order to analyse VEGFR2 phosphorylation in kidney and HUVEC homogenates was performed with the gel analysis tool from ImageJ. Signal intensities were measured by creation of a lane profile plot and measurement of the area with subtraction of the background signal. The ratio of phosphorylated VEGFR2 to total VEGFR2 protein was calculated and normalization to the control/untreated sample was conducted to directly compare different experiments.

Quantitative real-time PCR

Total RNA from murine kidneys was extracted with TRIzol® (Invitrogen) by homogenization with a Precellys homogenizer (Bertin technologies) at 5000 rpm. Chloroform was added and incubated on ice for 3 min. The samples were centrifuged at 12000 x*g* at 4°C for 15 min. 300 µl of the RNA phase were mixed with 300 µl isopropanol and centrifuged again at 12000 x*g* at 4°C for 10 min. The RNA pellet was washed two times with 70% Ethanol, air dried and reconstituted in RNAse-free H_2O. RNA concentration and quality was measured in a nanophotometer N60 (Implen). For cDNA synthesis, RNA was first digested with RQ1 DNAse (Promega) following reverse transcription with Maxima H minus reverse transcriptase (Thermo Fisher) according to the manufacturer's protocol. SyGreen Lo-ROX Mastermix (PCR Biosystems) with a Maxima Hot Start DNA polymerase (Thermo Scientific) were used for qPCR with a QuantStudio 3 thermocycler (Thermo Fisher). Sequences of qPCR primers (Sigma-Aldrich) are listed in Supplemental Table S3. Per sample four technical replicates were measured. Ct values were normalized to the two reference genes *Gapdh* and *Ppia*. Six biological replicates for each time point and genotype were analysed.

Cultivation of human umbilical vein endothelial cells (HUVECs)

Human umbilical vein endothelial cells (HUVECs) (#C2519A, Lonza) were cultivated in Endothelial Cell Growth Medium-2 supplemented with EGM^{TM}-2 SingleQuots Supplements (Lonza, CC-3162) at 37°C and 5% CO_2. For passaging, cells were detached with Tryp-LE Select (Gibco), counted, centrifuged at 250 x*g* for 10 min and seeded in 6 well plates. For all experiments cell passages from 2-6 were used.

Image acquisition

Stained sections from histological staining, *in situ* hybridization and BaseScope assay was documented with a Zeiss Axio Observer.Z1 microscope equipped with AxioCamMRm and AxioCamMRc digital cameras and an ApoTome module for structured illumination. Stained sections from indirect immunofluorescence were analyzed with the confocal laser scanning microscope ZEISS LSM 980 with Airyscan 2. Digital photographs were processed with the Zen 2012 software (Carl Zeiss Microscopy) or ImageJ. Cells were counted by visual inspection assisted by the interactive event counting tool of AxioVision and glomerular tuft areas were analysed with the manual contouring tool of the Zen 2012 software.

Single-cell RNA sequencing data processing

Single-cell RNA sequencing data was downloaded from the Gene Expression Omnibus portal under the accession number GSM4648414 (Naganuma, H. et al. (2021) Dev Biol 470, 62-73) or from www.kidneycellatlas.org and quality control of was performed using *scanpy* version 1.7.2 (Wolf, F. A. et al., (2018) Genome Biol 19, 15), which included filtering out cells with <200 genes detected and genes expressed in <3 cells. Clustering

analysis was performed, cell types were assigned using the genes mentioned in the original paper (Naganuma, H. et al. (2021) Dev Biol 470, 62-73) or already present in the public data, and were visualized using uniform manifold approximation and projection (UMAP) or t-distributed stochastic neighbor embedding (tSNE) dimensionality reduction. Differential gene expression analysis was done by normalizing the total raw counts per cell, *log* transforming the data and performing the comparison between *St8sia4* counts >0 to *St8sia4*=0 endothelial cells, using *scanpy*'s built-in functions. Data visualization of the expression of specific genes was performed using *scanpy*'s built-in plotting functions, including UMAP, tSNE and volcano plots.

Enzyme-linked immunosorbent assay

Half-area 96-well plates (Greiner REF 675101) were coated with 50 ng of purified E.coli K1 capsular polysaccharide (Schwarzer, D., et al., (2007) J Biol Chem 282, 2821-2831) in PBS overnight at 4°C. Non-specific binding sites were blocked with PBS/0.05% Tween (PBS-T) for 1 h at RT. After washing with PBS-T, serial dilutions of recombinant murine VEGF-A isoforms 120, 164 and 188 (Reliatech, M30-032, M30-004, M30-094) were applied for 1 h at RT. Subsequent incubations with anti-VEGF-A antibody and HRP-conjugated goat anti-rabbit IgG secondary antibody (see Supplemental Table S2) were performed for 1 h at RT in PBS-T. Colour development was performed with 3,3',5,5'-tetramethylbenzidine (Sigma) and H_2O_2. After 20 min reaction was stopped using 1 M H_2SO_4 and absorbance was measured at 450 nm (Biotek). Background signals obtained from wells lacking K1 capsular polysaccharide were subtracted from signals obtained from wells coated with K1 capsular polysaccharide prior to further analyses using Prism 9.

Supplemental Figures and Tables

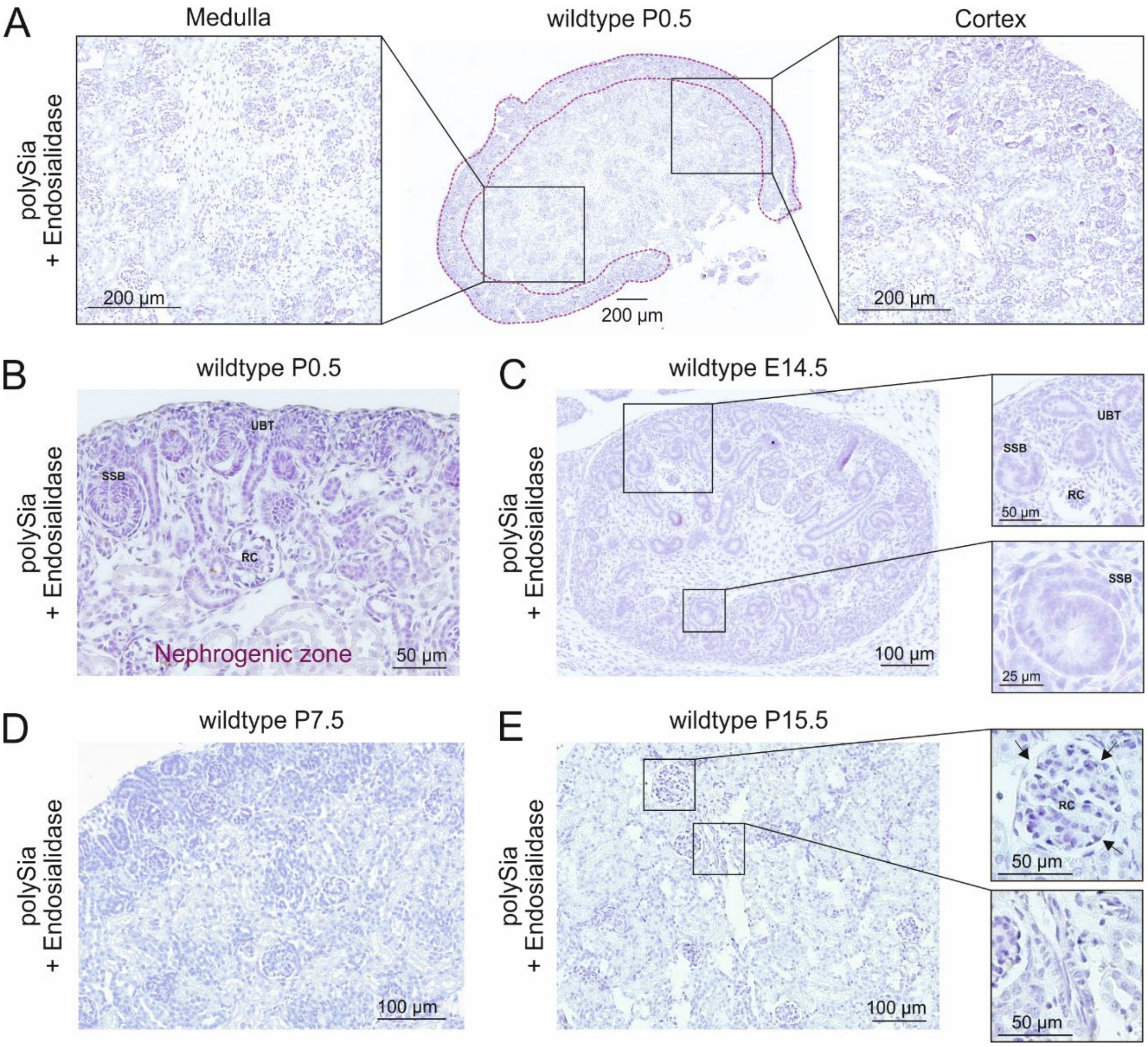

Supplemental Fig. S1. Specificity control of 735 immunohistochemistry on renal sections. (A) Consecutive renal section from wildtype mice at P0.5 from Fig. 1C was treated with endosialidase prior to the 735 staining procedure. The whole renal section is shown to appreciate complete loss of antibody staining with enlarged micrographs of the medullary and cortical area. Similarly, loss of 735 staining was observed in consecutive endosialidase treated renal sections from wildtype mice corresponding to **(B)** enlarged micrograph of the NZ at P0.5, where different stages of nephron development are visible, **(C-E)** consecutive renal section from wildtype mice at **(C)** E14.5 from Fig. 1C, **(D)** P7.5 from Fig. 1D and **(E)** P15.5 from Fig. 1E was treated with endosialidase prior to the 735 staining procedure. The whole renal section is shown to appreciate complete loss of antibody staining.

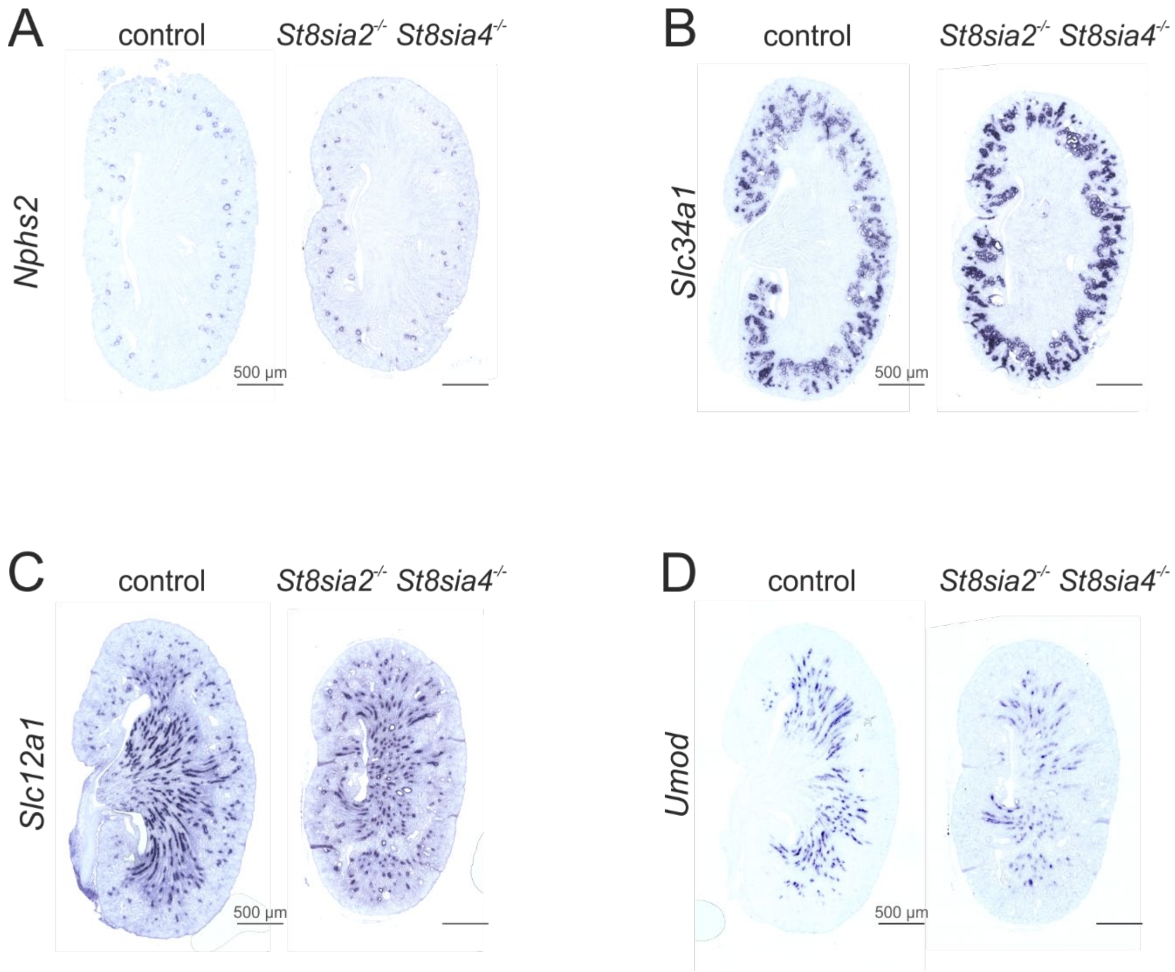

Supplemental Fig. S2. Kidney architecture markers in renal sections of wildtype and polySia-negative mice. *In situ* hybridization of renal architecture markers **(A)** *Nphs2* (Glomeruli), **(B)** *Slc34a1* (proximal tubules), **(C)** *Slc12a1* (distal tubules, thick ascending limbs), **(D)** *Umod* (distal tubules, collecting ducts) in newborn (P0.5) wildtype and *St8sia2*$^{-/-}$ *St8sia4*$^{-/-}$ mice. No obvious changes in marker expression were observed between the genotypes. Staining was performed on paraffin-embedded kidney sections as described in the methods section.

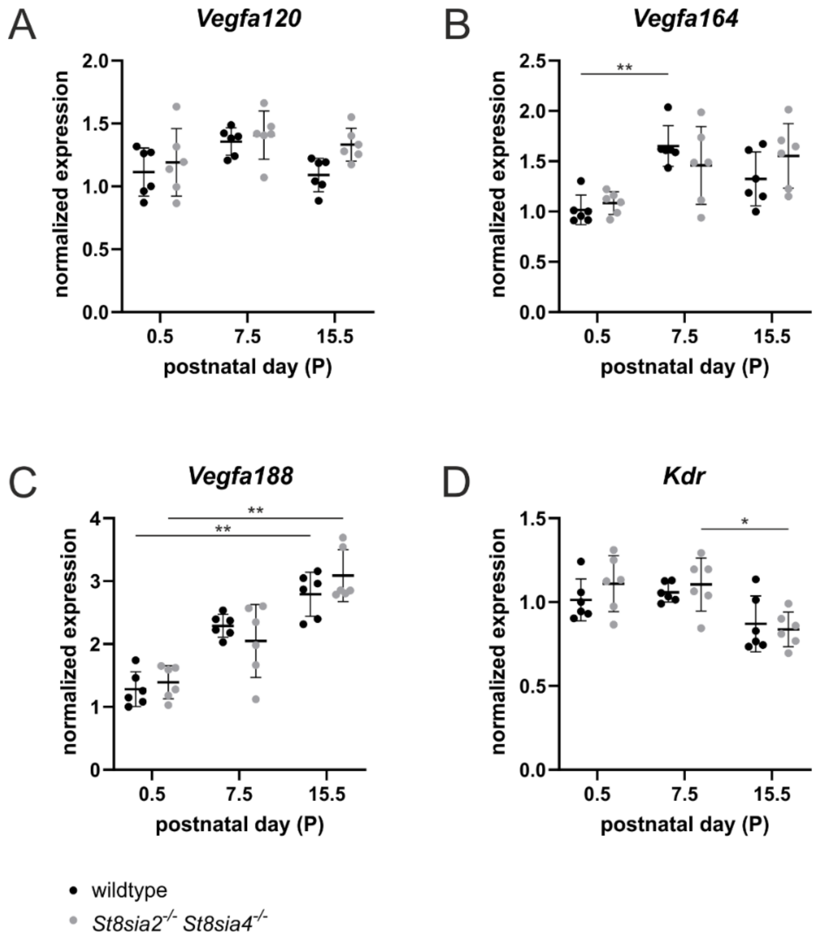

Supplemental Fig. S3. Relative mRNA expression of *Vegfa* isoforms and *Kdr* (VEGFR2) in murine kidney. Normalized gene expression of *Vegfa* isoforms and *Kdr* in wildtype (black) and *St8sia2*$^{-/-}$ *St8sia4*$^{-/-}$ (grey) kidneys at different postnatal (P) time points. n=6 biological samples were analyzed per time point and genotype. **(A)** Relative *Vegfa120* expression neither shows significant changes between the genotypes nor over time. **(B)** Relative *Vegfa164* expression increases slightly from P0.5 to P7.5. This increase is statistically significant ($p<0.05$) for wildtype but not for polySia-negative kidneys. **(C)** Relative *Vegfa188* expression increases in both genotypes from P0.5 to P15.5 and is statistically significant between P0.5 and P15.5 for both genotypes. **(D)** Relative *Kdr* (VEGFR2) expression decreases in both genotypes from P7.5 to P15.5 with statistical significance for polySia-negative samples. Statistical significance was determined by Kruskal-Wallis test and Dunn's multiple comparison, comparing genotypes at the same time points and each genotype over time. Statistical significance is indicated with asterisks (* $p<0.05$; ** $p<0.01$). Relative expression of all analyzed genes does not change significantly between the two genotypes.

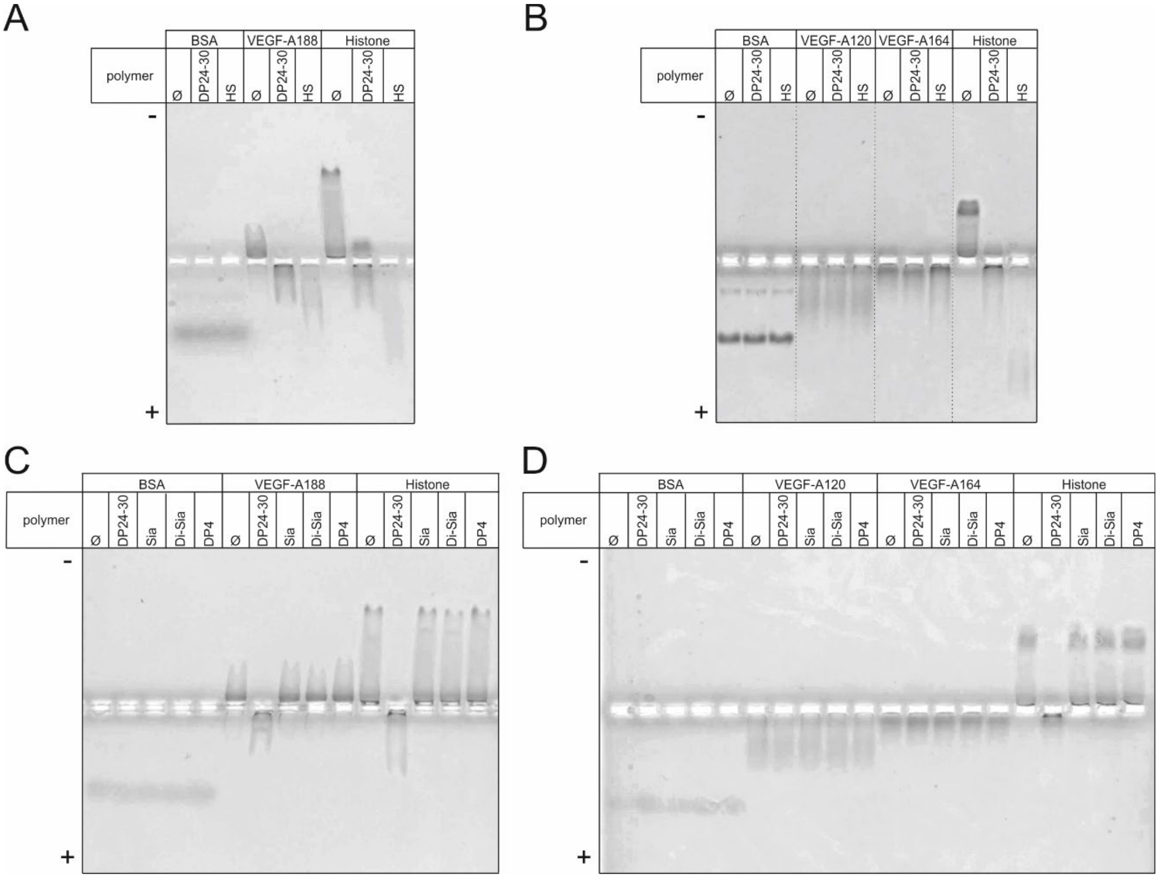

Supplemental Fig. S4. Binding assay of VEGF-A isoforms with polySia and HS as well as of mono-Sia, α2,8-linked di-Sia or oligo-Sia with DP4 in horizontal native PAGE. Bovine serum albumin (BSA, pI ~ 5) is shown as a negative control and histone as a known polySia and HS interaction partner is shown as a positive control (Mishra B et al., (2010). J Neurosci. 30(37):12400-13; Wildhagen KC et al. (2014) Blood. 123(7):1098-101). The binding of histone (pI = 11) to the polyanionic polysaccharides polySia or HS is indicated by an increased electrophoretic mobility of the bound protein towards the anode (+). **(A, B)** Analysis of **(A)** VEGF-A188 and **(B)** VEGF-A120 and -164 with polySia DP24-30 and HS. **(A)** Complex formation of VEGF-A188 (pI = 9.25) with both polySia and HS reverted the migration direction in horizontal native PAGE analysis at pH = 7.4 towards the anode (+), indicating direct binding of VEGF-A188 to both glycopolymers. **(B)** The mobility of BSA (pI ~ 5) and the VEGF-A120 (pI = 6.48) was not affected by polySia DP24-

30 or HS in native PAGE analysis at pH = 8.1. Upon interaction with HS, but not with polySia DP24-30, the electrophoretic mobility of VEGF-A164 (pI = 7.93) was increased towards the anode at pH = 8.1. **(C,D)** Binding analysis of **(C)** VEGF-A188 and **(D)** VEGF-A120 and VEGF-A164 to mono-Sia, α2,8-linked di-Sia or oligo-Sia with DP4 in horizontal native PAGE. Similar to the negative control BSA, incubation of all three VEGF-A isoforms with mono-Sia, α2,8-linked di-Sia or oligo-Sia with DP4 did not change the mobility of the proteins in native PAGE, indicating no complex formation. Binding was only observed for VEGF-A188 or histone with polySia DP24-30 in native PAGE analysis at pH = 7.4, indicating that the glycopolymer is required for complex formation. (+) anode; (-) cathode. Native PAGE conditions allowing optimal migration in PAGE were used for all three proteins.

.

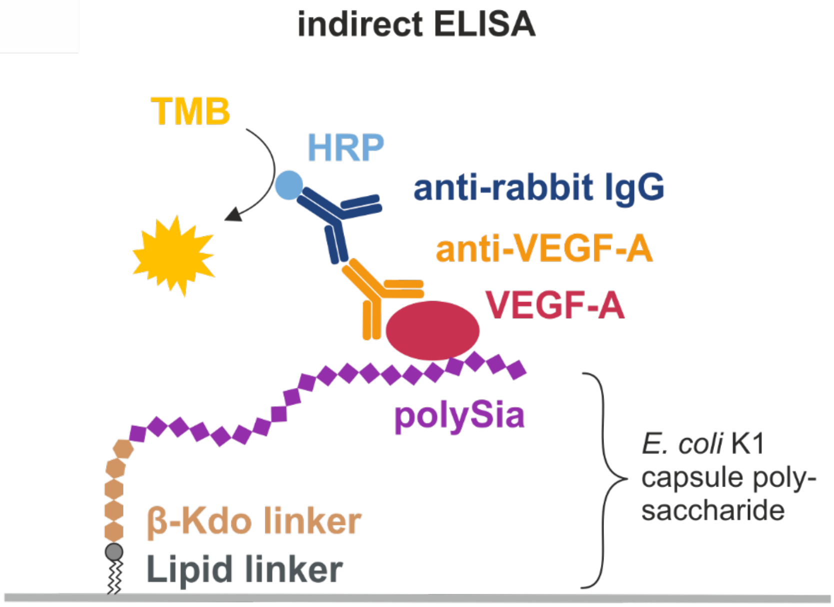

Supplemental Fig. S5. Scheme of the indirect ELISA setting to detect polySia interaction with VEGF-A. *E. coli* K1 capsule polysaccharide with avDP50 was immobilized and bound recombinant VEGF-A protein was detected with a pan anti-VEGF-A antibody and a secondary HRP-coupled anti-rabbit antibody. The substrate TMB was oxidized by HRP and absorption of the product was measured at 450 nm. β-Kdo: 3-Deoxy-β-d-manno-oct-2-ulosonic acid.

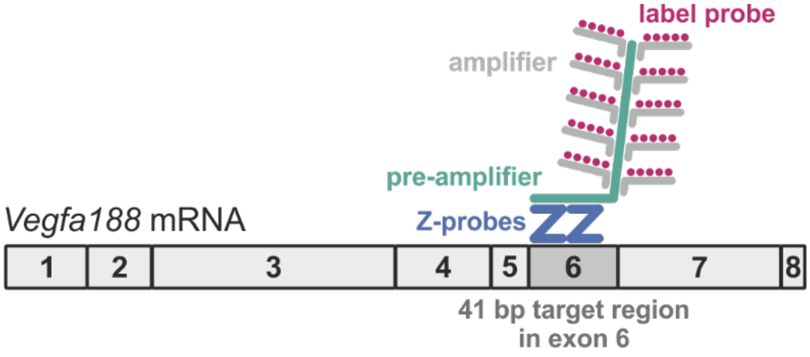

Supplemental Fig. S6. *Vegfa188* mRNA exon structure and illustration of the Base Scope assay principle. Exon 6 (72 bp), which is only present in the longest VEGF-A isoform 188, is recognized by a Z-probe pair (blue) detecting a target region of only 41 bp (nt 1441-1482 of NM_001287056.1). A pre-amplifier (green) binds to the Z-probe pair on the target mRNA and is detected by multiple amplifiers (grey). The labelled probes (red) bind to this amplification tree and are visualized by Fast RED staining (Figure 3 G-I).

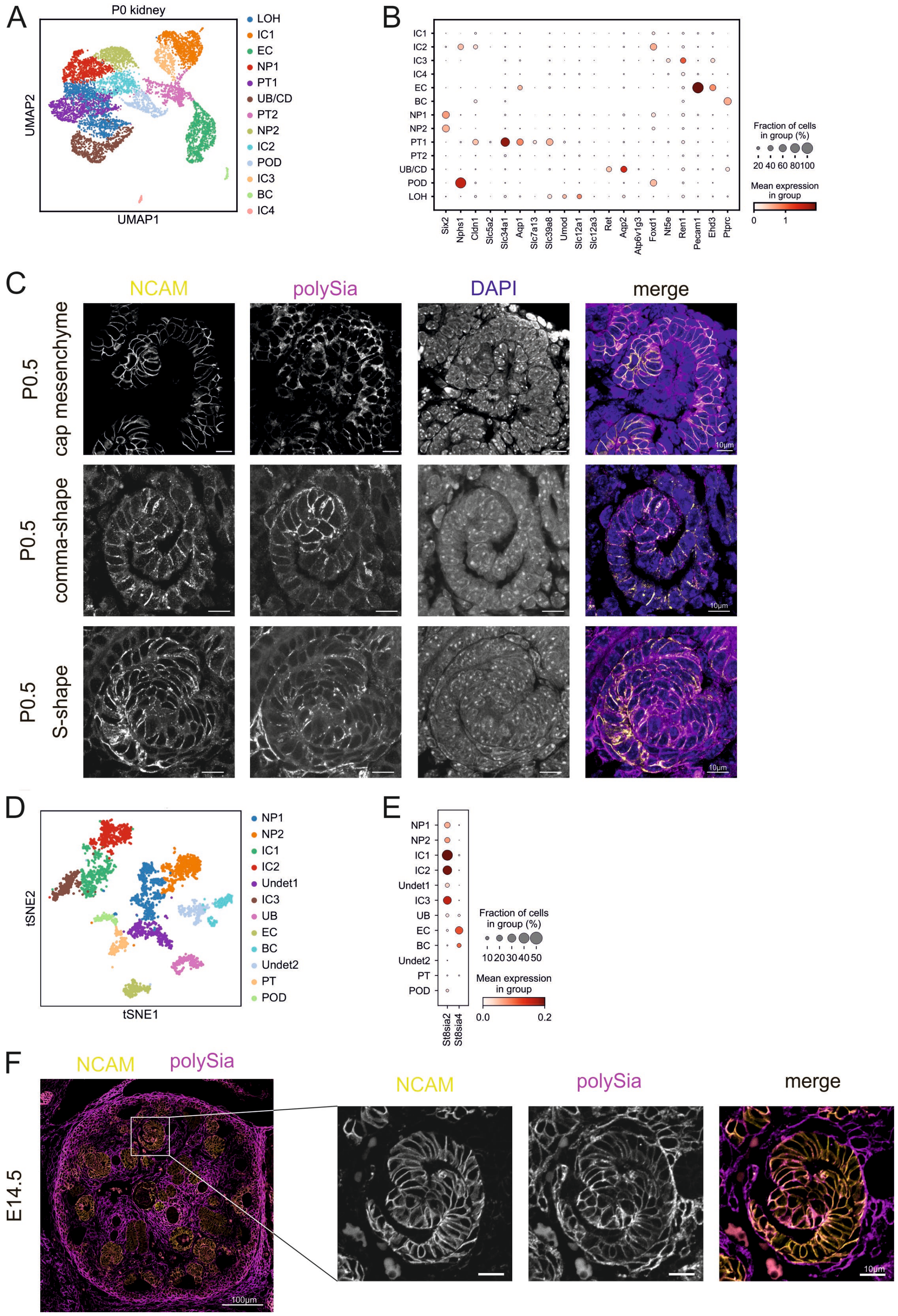

Supplemental Fig. S7. Polysialyltransferase and polySia expression in the nascent nephron. (A) Single cell RNA sequencing (scRNAseq) data processing. scRNAseq data

of P0 from C57BL/6N wildtype mice from Naganuma *et al.* (Naganuma, H. et al. (2021) Dev Biol 470, 62-73) was analyzed. **(A)** Clustering analysis was performed according to Naganuma *et al.* to identify cell types in leiden clusters. Thirteen different cell clusters were defined. **(B)** Differential expression of common cell type marker genes in the eiden cell clusters. BC: blood cell, EC: endothelial cell, IC: interstitial cell, LOH: loop of Henle, NP: nephron progenitor, POD: podocyte, PT: proximal tubule, UB: ureteric bud tip, CD: collecting duct. **(C)** Immunohistochemical co-staining of NCAM (yellow) and polySia (magenta) in renal sections from wildtype mice at P0.5. Three stages of nephron development, cap mesenchyme, comma- and S-shape bodies are shown, respectively. Cell nuclei were stained with DAPI (blue). Colocalisation of NCAM and polySia is visualized in white colour in the merged images. **(D)** ScRNAseq data of E15 wildtype kidney from Naganuma *et al.* (Naganuma, H. et al. (2021) Dev Biol 470, 62-73) was analyzed, and cell type annotation was done as with P0 data described in (A) **(E)** Evaluation of polysialyltransferase gene expression in different renal cell types (Leiden clusters) in E15 wildtype mice in the Naganuma dataset. **(F)** Immunohistochemical co-staining of NCAM (yellow) and polySia (magenta) in renal sections of wildtype mice at E14.5, with an enlarged micrograph of a developing nephron in an S-shaped body stage. Colocalisation of NCAM and polySia is visualized in white colour in the merged image. LOH: loop of Henle, IC: interstitial cell, EC: endothelial cell, NP: nephron progenitor, PT: proximal tubule, UB: ureteric bud tip, CD: collecting duct, POD: podocyte, BC: blood cell.

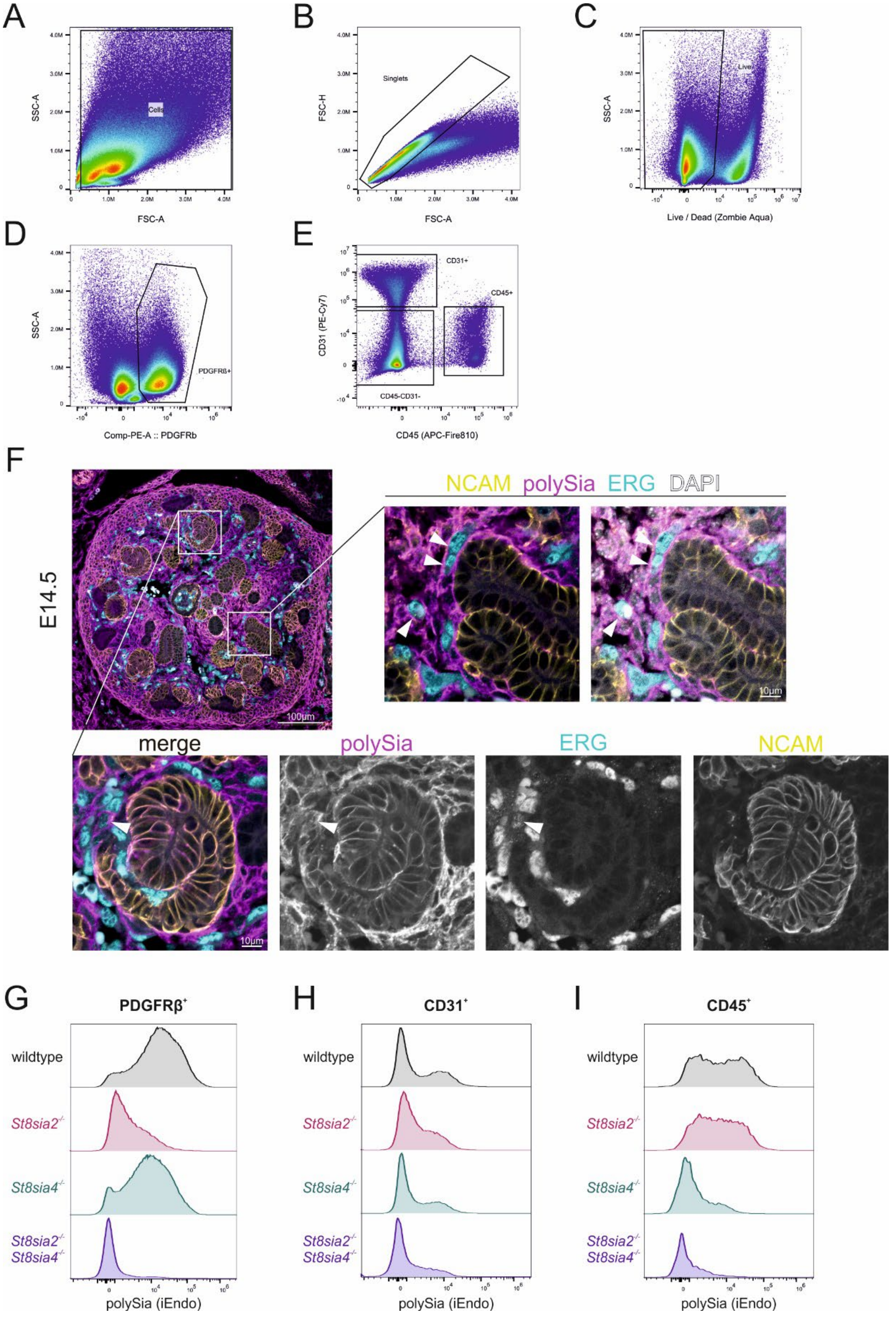

Supplemental Fig. S8. Spectral flow analysis of kidney single cell suspensions from newborn wildtype and polysialyltransferase knockout mouse strains *St8sia2*$^{-/-}$,

***St8sia4$^{-/-}$* and *St8sia2$^{-/-}$ St8sia4$^{-/-}$*. (A-E) Gating strategy for spectral flow cytometry analysis.** The displayed gating strategy was applied to spectral flow cytometry data of kidney single cell suspensions from P0 and P3 mice. Gating for **(A)** Cells, **(B)** cell singlets, **(C)** live cells, **(D)** PDGFRβ^{+} cells and **(E)** CD31^{+} and CD45^{+} cells was applied before analysis of iEndo staining. **(F)** Immunohistochemical co-staining for NCAM (yellow), polySia (magenta) and ERG as endothelial cell marker (cyan) in a renal section from wildtype kidney at E14.5, with a higher magnification of an comma-shaped body and renal epithelium. PolySia positive endothelial cells are depicted with white arrows. **(G-I)** Spectral flow cytometry analysis was conducted with renal single cell suspensions from newborn wildtype and polysialyltransferase knockout mouse strains *St8sia2$^{-/-}$, St8sia4$^{-/-}$ and St8sia2$^{-/-}$ St8sia4$^{-/-}$.* Cell suspensions were stained for polySia with inactive endosialidase (iEndo) and for **(G)** interstitial cells (PDGFRβ), **(H)** endothelial cells (CD31) and **(I)** immune cells (CD45) to discriminate between different cell types**.** Normalized histograms from each one biological replicate are depicted exemplarily.

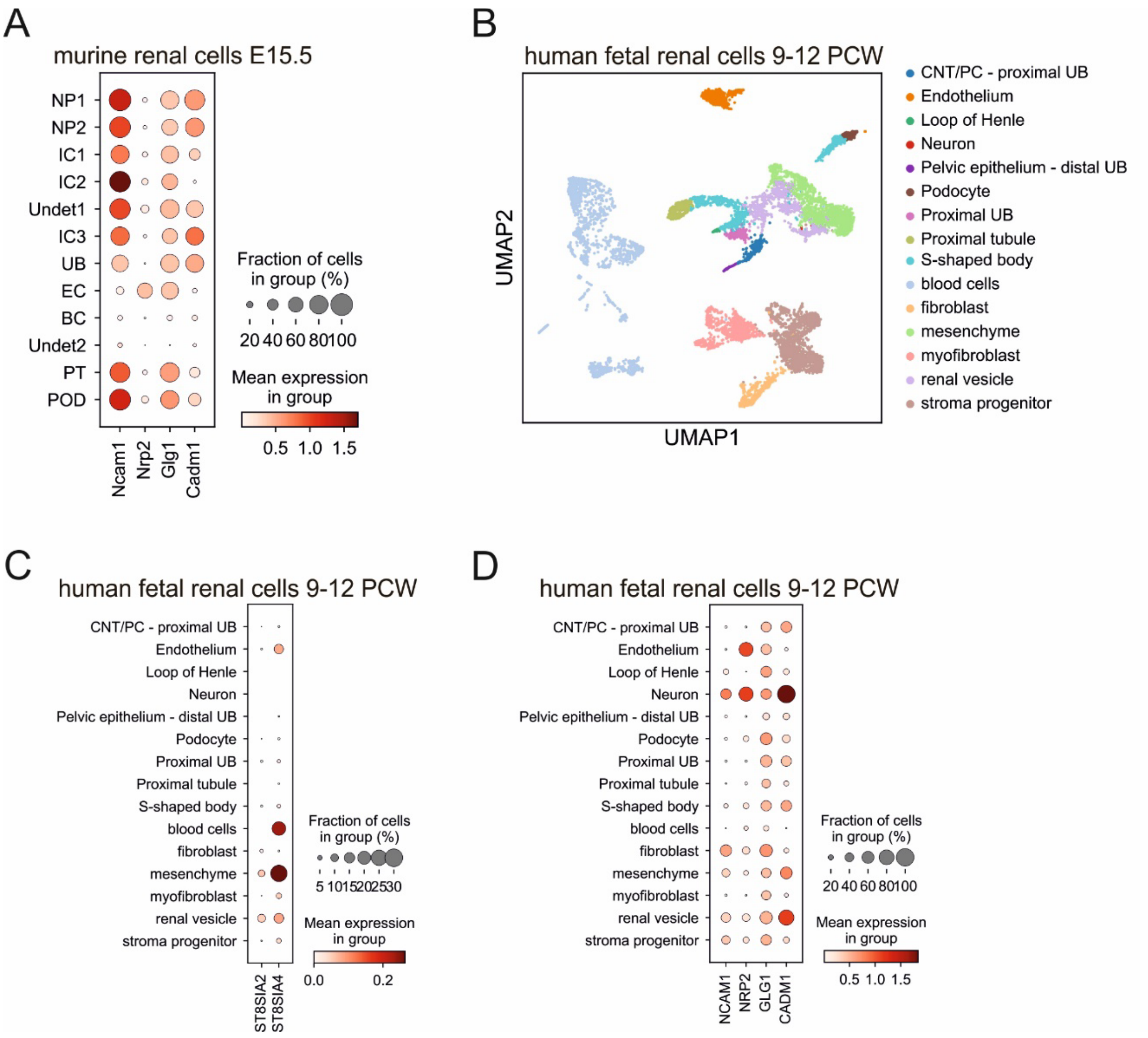

Supplemental Fig. S9. Single Cell RNAseq analysis of mouse and human renal samples during development

(A) Evaluation of gene expression values of known polySia protein carriers in different renal cell types (Leiden clusters) in murine E15 kidney samples from Naganuma *et al*. according to Supplemental Fig. S7D. **(B)** ScRNAseq data of post-conception week 9-12 human kidney samples from Stewart *et al*. (Stewart et al. (2019) Science 365(6460), 1461-1466). Clustering and cell type annotation was extracted from the publicly available files. **(C)** Evaluation of polysialyltransferase gene expression in different renal cell types from the human fetal kidney dataset (9-12 weeks) described in (B). Polysialyltransferase

expression in blood cells, interstitial cells, renal progenitor cells and endothelial cells is conserved in mouse and human. **(D)** Evaluation of gene expression values of known polySia protein carriers in different annotated renal cell types in human kidney samples at week 9-12 described in (B). LOH: loop of Henle, IC: interstitial cell, EC: endothelial cell, NP: nephron progenitor, PT: proximal tubule, UB: ureteric bud tip, CD: collecting duct, POD: podocyte, BC: blood cell. *Ncam1* or NCAM1: neural cell adhesion molecule, *Nrp2* or *NRP2*: neuropilin-2, *Glg1* or *GLG1*: E-selectin ligand-1/Golgi Glycoprotein 1, *Cadm1* or *CADM1*: SynCAM 1/Cell adhesion molecule 1.

Supplemental Table S1. Genotyping primer

Primer name	Gene	Sequence (5` to 3`)
STX F13	*St8sia2*	AACGCTTTACCAACTGTGCTATCT
STX B8	*St8sia2*	ATGTCTGGAAAGGAATGGATGTCT
STX B14	*St8sia2*	TTGGGGTCTTGTGGTAAATCAGTC
LW 13	*St8sia4*	CTCAGTTCTGGCTATTTCTTTTGT
LW 14	*St8sia4*	GAGCTCACAACGACTCTCCGAGC
LW 18	*St8sia4*	ACCGCGAGGCGGTTTTCTCCGGC
NCAM I	*Ncam*	CTGCCTCAGATAGTGACCCAGTGC
NCAM II	*Ncam*	CGGAGAACCTGCGTGCAATCCATC
NCAM III	*Ncam*	TTGGAGGCAGGGAGCTGACCACAT

Supplemental Table S2. Antibodies

Primary antibody	Source	Manufacturer	Catalog no./ Reference	Dilution
735 (polySia)	mouse	Genovac	Frosch *et al.* (1985)	1:1,000-3,000 (WB)<br>1:500 (IF/IHC)
β-ACTIN	mouse	Millipore	MAB1501	1:100,000 (WB)
CD31	rabbit	SinoBiological	SB50408-T16	1:10.000 (IHC)
CD31-PE-Cy7	rat	Biolegend	102523	1:200 (FC)
CD45-APC/Fire 810	rat	Biolegend	103173	1:300 (FC)
ERG	rabbit	abcam	Ab92513	1:3,000 (IHC)
GATA3	rabbit	Cell Signaling	5852	1:50 (IHC)
NCAM (OB11)	mouse	Sigma	C-9672	1:100 (IF)
NCAM-APC	rat	R & D Systems	FAB7820A-025UG	1:200 (FC)
PDGFRβ-PE	rat	Biolegend	136005	1:100 (FC)
VEGF-A	rabbit	abcam	Ab46154	1:1,000 (WB)<br>1:10,000 (ELISA)<br>1:500 (IF)
VEGFR2	rabbit	Cell Signaling	D5B1 (#9698)	1:2,000 (WB)<br>1:500 (IF)
VEGFR2-P (phosphoTyr1175)	rabbit	Cell Signaling	D5B11 (#37705)	1:2,000 (WB)
WT1	rabbit	abcam	ab89901	1:200 (IHC)
**Secondary antibody**				
mouse-HRP	goat	Southern Biotech	1010-05	1:15,000 (WB)<br>1:500 (IHC)
rabbit-IgG-HRP	goat	Jackson	111-035-003	1:15,000 (WB)<br>1:10,000 (ELISA)
rabbit-IgG-HRP (ImmPRESS)	goat	Vector	MP-7451	Undiluted (IHC)
mouse-IgG-AlexaFluor647	goat	Molecular Probes	A21236	1:500 (IF)
mouse-IgG1-AlexaFluor488	goat	Molecular Probes	A21121	1:500 (IF)
mouse-IgG2a-AlexaFluor647	goat	Molecular Probes	A21241	1:500 (IF)
rabbit-IgG-AlexaFluor488	donkey	Molecular probes	A21206	1:200 (FC)

rabbit-IgG-AlexaFluor555	donkey	Invitrogen	A31572	1:500 (IF)
rabbit-IgG-AlexaFluor568	goat	Molecular Probes	A11036	1:500 (IF)
Streptavidin-AlexaFluor647	-	Invitrogen	S21374	1:500 (FC)
Streptavidin-SparkUV387	-	Biolegend	405351	1:500 (FC)

WB (Western Blotting), IHC (Immunohistochemistry), IF (Indirect immunofluorescence), FC (Flow cytometry); ELISA (enzyme-linked immunosorbent assay)

Frosch M. et al. (1985) Proc Natl Acad Sci U S A. 82(4):1194-8.

Supplemental Table S3. Quantitative real-time PCR primer. *Vegfa* isoform specific primer sequences according to Tillo, M. *et al.* (2015) Development 15;142(2):314-9.

Gene	Sequence (5`to 3`)
Vegfa120-Fw	GTAACGATGAAGCCCTGGAG
Vegfa120-Rev	CCTTGGCTTGTCACATTTTTC
Vegfa164-Fw	CAGAACAAAGCCAGAAAATCAC
Vegfa164-Rev	GCCTTGGCTTGTCACATCT
Vegfa188-Fw	AGTTCGAGGAAAGGGAAAGG
Vegfa188-Rev	GCCTTGGCTTGTCACATCT
Kdr-Fw	GACTCTCCCTGCCTACCTCA
Kdr-Rev	CTGTCTGGCTGTCATCTGGG
Gapdh-Fw	GTGATGGGTGTGAACCACGA
Gapdh-Rev	TGGCAGTGATGGCATGGACT
Ppia-Fw	CCACAGTCGGAAATGGTGAT
Ppia-Rev	TGCACTGCCAAGACTGAATG
